# CTCF-RNA interactions orchestrate cell-specific chromatin loop organization

**DOI:** 10.1126/sciadv.ady5507

**Published:** 2025-11-26

**Authors:** Kimberly Lucero, Sungwook Han, Pin-Yao Huang, Xiang Qiu, Esteban O. Mazzoni, Danny Reinberg

**Affiliations:** ^1^ Department of Cell Biology and Regenerative Medicine, New York University Langone Medical Center, New York, NY, USA.; ^2^ Department of Biochemistry and Molecular Pharmacology, New York University Langone Medical Center, New York, NY, USA.; ^3^ Sylvester Comprehensive Cancer Center, Department of Human Genetics, University of Miami Miller School of Medicine, Miami, FL, USA.; ^4^ Howard Hughes Medical Institute, University of Miami Miller School of Medicine, Miami, FL, USA.

## Abstract

CCCTC-binding factor (CTCF) is essential for chromatin organization. CTCF interacts with endogenous RNAs, and deletion of its ZF1 RNA binding region (∆ZF1) disrupts chromatin loops in mouse embryonic stem cells (ESCs). However, the functional significance of CTCF-ZF1 RNA interactions during cell differentiation is unknown. Using an ESC–to–neural progenitor cell (NPC) differentiation model, we show that CTCF-ZF1 is crucial for maintaining cell type–specific chromatin loops. Expression of CTCF-∆ZF1 leads to disrupted loops and dysregulation of genes within these loops, particularly those involved in neuronal development and function. We identified NPC-specific, CTCF-ZF1 interacting RNAs. Truncation of two such coding RNAs, *Podxl* and *Grb10*, disrupted chromatin loops in cis, similar to the disruption seen in CTCF-∆ZF1–expressing NPCs. These findings underscore the inherent importance of CTCF-ZF1 RNA interactions in preserving cell-specific genome structure and cellular identity.

## Teaser

During NPC differentiation, up-regulated *Podxl* and *Grb10* RNAs interact with CTCF-ZF1 to facilitate chromatin loop formation.

## INTRODUCTION

One of the long-standing questions in biology is how a single cell can give rise to multiple cell types. Despite having the same genome, gene expression varies from one cell type to another, resulting in the diverse tissues that comprise a multicellular organism. The nonrandom structural organization of the genome is crucial for maintaining cell type–specific transcriptional programs that establish identity and proper function (*1*). Disruption of chromatin structure can lead to oncogenesis (*2*) or developmental abnormalities (*3*–*5*).

The structure of the genome is organized hierarchically. Interphase chromosomes occupy well-defined territories within the nuclear space (*6*). Within these chromosome territories are A and B compartments, corresponding to euchromatin and heterochromatin, respectively (*7*). Within compartments are fundamental structures, called topologically associating domains (TADs), which are defined as contiguous chromatin that is more frequently interacting with itself compared to chromatin outside the TAD (*8*, *9*). TADs are insulated regions of chromatin, which are composed of either actively transcribed regions or silent chromatin domains (*10*). Last, colocalized at or within TAD boundaries are chromatin loops, defined as genomic regions that are in closer physical proximity to each other than to their intervening sequences (*11*). TAD boundaries that are demarcated by chromatin loops occur frequently and are referred to as “loop domains” (*11*).

Central to the structural maintenance of chromatin loops (as well as TADs) in the mammalian system is CCCTC-binding factor (CTCF) (*12*), which is ubiquitously expressed and binds with high affinity to a conserved DNA motif (*13*). CTCF binding sites are enriched at most chromatin loop anchors (*11*) and are important for maintaining chromatin loops (*12*). Chromatin loops bound by CTCF at their base (“CTCF loop anchors”) are frequently found in close proximity to genomic regions enriched for active histone modifications, RNA polymerase II, and transcription start sites (TSSs) (*14*). These anchors play multifaceted and context-specific roles in transcriptional regulation by (i) bringing promoters close to distal enhancers to promote transcription (*15*), (ii) preventing ectopic contacts between promoters and enhancers not within the same loop domain (*3*, *10*), and (iii) acting as insulators or barriers to the spreading of active histone modifications (*4*).

The formation of CTCF loop anchors involves CTCF interaction with the cohesin complex, composed of Rad21, Smc3, Smc1a, and Stag1/Stag2 (*16*, *17*). Chromatin loops form apparently through a loop extrusion mechanism, whereby cohesin tethers two regions together and extrudes chromatin until it contacts two distant CTCF-bound sites in convergent orientation (*18*–*21*). Cohesin accessory proteins, such as Nipbl-Mau2, Wapl, Pds5a, and Pds5b, regulate cohesin loading, unloading, and processivity, thus modulating chromatin loop formation (*22*–*24*).

Although the loop extrusion model is an elegant and widely accepted explanation for how chromatin loops are formed, some observations regarding chromatin loops remain unexplained. Although most chromatin loops are bound by CTCF, there are more CTCF binding sites than loop anchors, and most CTCF binding sites are not at chromatin loops (*8*, *11*). It remains unclear why certain CTCF binding sites function as loop anchors whereas others function in transcription. In addition, CTCF loop anchors are dynamic and can change upon differentiation, such as during the differentiation of mouse embryonic stem cells (ESCs) into neural progenitor cells (NPCs) (*25*). The sites to which CTCF actively binds are largely invariant between cell types (*26*), yet they exhibit differential chromatin interactions. Thus, the reorganization of CTCF loop anchors upon differentiation is not well understood and likely involves cell-specific regulatory elements that cooperate with CTCF. It was previously found that, in addition to CTCF, other proteins such as Maz, Patz1, and Znf263 modulate insulation and gene expression in a cell-specific manner (*27*–*29*).

In this study, we examined the role of cell-specific CTCF-RNA interactions in chromatin loop formation based on the following evidence. CTCF comprises a central zinc finger domain with 11 zinc fingers (ZFs). The central ZFs 3 to 7 directly interact with the 15–base pair (bp) core DNA motif and are crucial for CTCF DNA binding (*30*). The peripheral ZF1, ZF10, and a C-terminal region (RBRi) are RNA binding domains (*31*–*34*). CTCF interacts with thousands of endogenous RNAs (*35*). Notably, a deletion in ZF1 of CTCF (∆ZF1) thwarts the integrity of some chromatin loops in vivo (*33*). Yet, deletion or point mutation in ZF1 does not affect CTCF DNA binding activity in vitro (*36*) and gives rise to a modest loss in vivo (*30*). A deletion in ZF10 of CTCF (∆ZF10) also results in decreased integrity of chromatin loops, albeit to a much lesser degree compared to ΔZF1 (*31*). ∆ZF1 and ∆ZF10 mutants display defects in self-association compared to wild type (WT) (*31*), which may explain the observed loss of chromatin loops. Similarly, a deletion in RBRi (ΔRBRi) leads to loss of chromatin loops (*33*), as well as decreased CTCF protein self-clustering in vitro (*32*) and in vivo (*33*). In addition to its role in facilitating CTCF-CTCF interactions, RNA has been shown to either facilitate or decrease CTCF binding to chromatin and thereby influence chromatin architecture and transcription (*35*, *37*–*39*).

Given the evidence above, we focused on elucidating the broader role and importance of CTCF-ZF1 in chromatin loop maintenance. Using an in vitro model system of ESC to NPC differentiation, we sought to answer the following questions: (i) Are CTCF-ZF1 RNA interactions required for the maintenance of cell type–specific chromatin loops? (ii) How does the ∆ZF1 mutation affect gene expression within these loops? (iii) Are there cell type–specific CTCF-ZF1 RNA interactions, and if so, are they important in maintaining cell type–specific chromatin loops? Using a CTCF-AID2 degron system coupled with rescue experiments involving doxycycline (dox)–inducible CTCF, either WT or ∆ZF1, we uncovered a vital role of CTCF-RNA interactions in chromatin loop organization. Our findings highlight that CTCF-ZF1 interactions with RNA are central to the integrity of cell-specific genome structure and cellular identity.

## RESULTS

### Acute CTCF depletion and rescue using the AID2 system

To determine the role of CTCF-ZF1 in genome organization, we generated mouse ESC lines (ES-E14TG2a) with an endogenous, homozygous deletion of CTCF-ZF1 (amino acids 264 to 277; ΔZF1) (fig. S1, A and B). The ΔZF1 ESCs had similar CTCF and Rad21 protein levels as WT but exhibited decreased protein levels of the pluripotency gene Oct3/4 (fig. S1C), suggesting that the mutant would have differentiation defects. We differentiated the ΔZF1 ESCs into NPCs following an established protocol (*40*) and assessed differentiation efficiency by immunostaining with Sox1, an NPC-specific marker (*40*). The ΔZF1 mutant exhibited inefficient differentiation, with a reduced percentage (fig. S1D) and total count of Sox1+ cells (fig. S1E), indicating that the CTCF-ZF1 region is crucial for cellular differentiation.

**Fig. 1.**
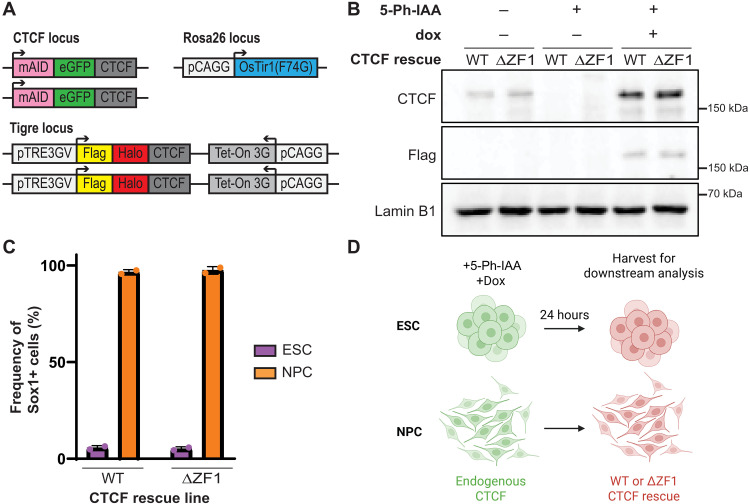
Acute CTCF depletion and rescue using the AID2 system. (**A**) Schematic showing the CTCF-AID2 degron and dox-inducible rescue system generated in ESCs. (**B**) Western blot of CTCF-AID2 and rescue lines after 24 hours with no treatment or treatment with 5-Ph-IAA or 5-Ph-IAA and dox. (**C**) CTCF-AID2 and dox-inducible rescue lines were differentiated into NPCs for 2 days. Cells were immunostained with anti-Sox1 antibody and percentages of Sox1+ cells were quantified by flow cytometry analysis. Neither 5-Ph-IAA nor dox was added. Data are represented as means ± SEM, *N* = 2 biological replicates. (**D**) Diagram of the experimental approach. ESCs or NPCs were treated with 5-Ph-IAA and dox and then harvested for downstream analysis after 24 hours. Image created in BioRender. Lucero, K. (2025) (https://BioRender.com/hu9z2jx).

Given the differentiation challenges with ESCs expressing ΔZF1 constitutively, we instead developed ESC lines having an AID2 degron system for CTCF, coupled with dox-inducible CTCF rescues (Fig. 1A and fig. S2A). This system allows for the degradation of endogenous CTCF and the inducible expression of tagged rescue CTCF variants, either WT or ΔZF1, in a temporally controlled manner postdifferentiation into NPCs.

To generate the CTCF-AID2 degron in the ES-E14TG2a background, we used CRISPR-Cas9–based gene editing to tag both endogenous CTCF alleles with mini-auxin inducible degron–enhanced green fluorescent protein (mAID-eGFP) at the N terminus. We then inserted OsTir1(F74G) into the Rosa26 locus to facilitate inducible degradation. OsTir1(F74G) interacts with mAID in the presence of the auxin analog 5-Ph-IAA, leading to ubiquitination by E3 ligase and subsequent proteasome-dependent degradation of mAID-eGFP–tagged CTCF (*41*). We then integrated dox-inducible Flag-Halo–tagged versions of CTCF, either WT (control) or ∆ZF1 into the Tigre locus of the CTCF-AID2 degron line.

First, we assessed the timing of CTCF degradation in ESCs using eGFP fluorescence (mAID-eGFP-CTCF). As expected, 100% of CTCF-AID2 cells strongly expressed eGFP, and the addition of 5-Ph-IAA led to a near-complete loss of eGFP fluorescence within 1 hour (fig. S2B). Next, we tested the efficiency of rescue expression by conjugating the Halo portion of the tagged CTCF to a JF646-fluorescent HaloTag. Almost 100% of cells expressed CTCF, either WT or ∆ZF1, at 24 hours after dox treatment (fig. S2C). Western blot analysis also confirmed degradation of mAID-eGFP-CTCF and showed similar Flag-Halo-CTCF protein levels between the WT and ΔZF1 rescues (Fig. 1B). We verified that the WT and ΔZF1 rescue lines expressing tagged endogenous CTCF (without 5-Ph-IAA or dox treatment) showed Sox1 expression in over 90% of cells by day 2 of NPC differentiation (Fig. 1C). For the experimental setup, we maintained ESCs in culture while differentiating the same lines into NPCs in parallel. ESCs and NPCs were then treated with 5-Ph-IAA and dox and harvested after 24 hours for analysis (Fig. 1D). We limited the rescue expression to 24 hours to capture more direct structural effects and minimize indirect effects from cascading gene expression changes due to mutant expression. Using this CTCF-AID2 and dox-inducible rescue system, we next investigated the role of CTCF-ZF1 RNA interactions in both differentiated NPCs and their parental ESCs.

### CTCF loop anchors induced after differentiation are disrupted in CTCF-ΔZF1

**Fig. 2.**
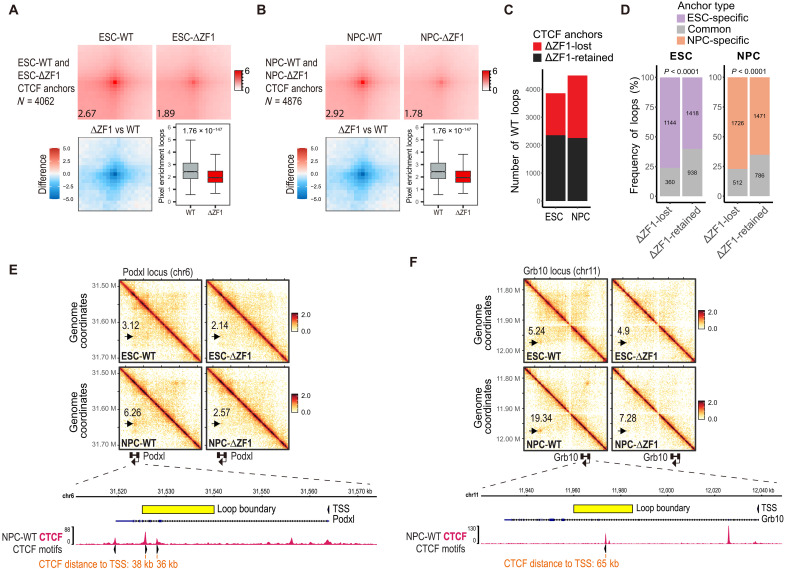
CTCF loop anchors induced after differentiation are disrupted in the ΔZF1 mutant. (**A** and **B**) APA plots (top) of CTCF loop anchors in (A) ESCs and (B) NPCs, comparing WT and ΔZF1. APA was generated using the combined set of CTCF loop anchors called in both WT and ΔZF1. The numbers indicate the APA score calculated using Juicer. The bottom-left panel is the difference in APA enrichment between ΔZF1 and WT. The bottom-right panel is a box plot quantifying the pixel enrichment of each loop. p-values were determined using the Wilcoxon signed-rank test. Plots represent merged biological replicates (*N* = 2). (**C**) Bar plots showing the number of WT CTCF loop anchors and the proportions that are lost or retained in the ΔZF1 mutant. Loops were classified as ΔZF1-lost if they were found to be significant only in WT but not in ΔZF1 (*q* value cutoff of 0.01) and if contact counts had log_2_ fold change ≤ −1 (see Materials and Methods). (**D**) Bar plot showing the proportions of cell type–specific and common loops among ΔZF1-lost or ΔZF1-retained loops. The numbers indicate the absolute numbers of loops. *P* values were determined using Fisher’s exact test. (**E** and **F**) Micro-C contact heatmaps (top) at the (E) *Podxl* and (F) *Grb10* loci. Arrows point to CTCF-bound loops. Numbers above the arrows indicate the fold enrichment of pixel intensity at the indicated loop anchor relative to the local background, as calculated using Cooltools. (Bottom) Loop anchor annotation and CTCF ChIP-seq track at the gene body of (E) *Podxl* and (F) *Grb10*. CTCF motifs with their orientation are annotated. The distance of the CTCF binding sites to the gene TSS is indicated. The data are represented as merged biological replicates (Micro-C *N* = 2; CTCF ChIP-seq *N* = 4).

To determine the role of CTCF-ZF1 on genome organization, we first assessed the impact of the ΔZF1 mutation on loop anchors in ESCs and NPCs expressing the ΔZF1 mutant rescue (ESC-ΔZF1 and NPC-ΔZF1, respectively). We performed Micro-C (*42*) and used FitHiC2 (*43*) to identify chromatin loops at a 5-kb resolution [false discovery rate (FDR) ≤ 0.01]. Replicate reproducibility was confirmed using HiCRep (*44*), which showed high stratum-adjusted correlation coefficients (SCC > 0.9) across all chromosomes (fig. S3, A and B). To specifically identify CTCF loop anchors, we performed CTCF chromatin immunoprecipitation sequencing (ChIP-seq) to determine CTCF binding sites and overlapped called ChIP-seq peaks with chromatin loop boundaries. We then evaluated contact strength at both CTCF-bound and non–CTCF-bound loop anchors. Although a slight reduction in contact counts was observed at non–CTCF-bound anchors—suggesting indirect effects on chromatin structure—the reduction at CTCF-bound anchors was substantially greater and highly significant (fig. S3, C and D), supporting a direct and specific role for the CTCF-ZF1 region in stabilizing chromatin loops. To assess direct effects, our subsequent analyses focused on CTCF-bound loop anchors (CTCF anchors) in both WT and ∆ZF1. We performed aggregate peak analysis (APA) to quantify the average interaction signal across all CTCF anchors (*45*). Consistent with previous findings (*31*), we observed a decrease in CTCF anchors in ESC-ΔZF1 (Fig. 2A). A similar reduction was also observed in NPC-ΔZF1 (Fig. 2B). CTCF loop anchors were independently called in both WT and ΔZF1. Venn diagrams comparing the two conditions reveal 202 gained anchors in ESCs and 381 in NPCs with the ΔZF1 mutation (fig. S3, E and F). However, these gains are relatively modest compared to the substantial number of lost anchors—1504 of 3860 (40%) in ESCs and 2238 of 4495 (50%) in NPCs—highlighting loop loss as the predominant phenotype (Fig. 2C).

To further characterize ZF1-dependent (ΔZF1-lost) versus ZF1-independent (ΔZF1-retained) CTCF loops, we performed a comparative analysis of cohesin and TAD colocalization, loop size, transcriptional activity, and chromatin state (fig. S3, G to J). Both loop types were bound by cohesin (Smc3) and were colocalized at TAD boundaries at similarly high frequencies (fig. S3, G and H), suggesting that colocalization with cohesin or TADs alone does not account for ZF1 dependence. Notably, ZF1-dependent loops were significantly longer in genomic span than ZF1-independent loops (fig. S3I), consistent with the idea that longer-range interactions—with inherently lower contact probabilities—may rely more on RNA-mediated stabilization, whereas shorter loops can be maintained by direct protein-DNA or protein-protein interactions.

To assess whether chromatin activity or transcriptional output underlies ZF1 dependence, we analyzed RNA sequencing (RNA-seq) data (this study), assay for transposase-accessible chromatin using sequencing (ATAC-seq) data (*46*), and ChIP-seq profiles for histone modifications—including H3K27ac, H3K4me3, H3K27me3, and H3K9me3 (*47*)—at ESC loop anchors. We found no significant differences in the enrichment of active chromatin marks (H3K27ac and H3K4me3), chromatin accessibility (ATAC-seq), or transcriptional activity (RNA-seq) between ZF1-dependent and ZF1-independent loops (fig. S3J). Repressive histone marks (H3K27me3 and H3K9me3) also showed no notable differences (fig. S3J). These results suggest that ZF1 dependence is not simply associated with changes in enhancer activity, promoter usage, or transcription but may instead reflect more specific functional features of CTCF-RNA interactions.

We then evaluated the impact of ΔZF1 on higher-order chromatin organization. We used principal components analysis (PCA) to identify the spatial segregation of A and B compartments, corresponding to positive and negative values of the first eigenvector, respectively (*7*). Our analysis revealed that the compartments in ESC-ΔZF1 and NPC-ΔZF1 were largely similar to those in WT cells, with only ~3% of compartments showing a shift from A-to-B or B-to-A (fig. S4A). We found a high correlation between the first eigenvectors of WT and ΔZF1 (*R*^2^ = 0.98) (fig. S4, B and C) and the absence of noticeable changes on the plaid pattern observed in Micro-C heatmaps (fig. S4, D and E). These results are consistent with previous studies showing that compartmentalization is CTCF independent (*12*, *17*).

Chromatin loops are frequently colocalized at TAD boundaries, and the loop extrusion model proposes that TADs emerge from multiple dynamically formed loops during extrusion (*18*, *19*). Given the widespread reduction in loops, we investigated whether TADs were also affected in ΔZF1. We used Arrowhead (*45*) to identify TADs and performed aggregate TAD analysis (ATA) (*48*) to average the results of all TADs. Comparison of ATA between WT and ΔZF1 revealed a decrease in long-range interactions within TADs in ΔZF1 (fig. S5, A and B). The decrease in interactions was most prominently observed at TAD boundaries (fig. S5, A and B). To compare the level of interaction separation at TAD boundaries, we calculated diamond insulation scores (*49*), which quantify the degree of interactions with neighboring regions. A lower insulation score indicates a stronger TAD boundary. We plotted the insulation score profiles for WT and ΔZF1 centered on CTCF ChIP-seq peaks and observed an increase in insulation scores in ΔZF1, indicating weakened TAD boundaries (fig. S5 C and D). These results show that the CTCF-ZF1 RNA binding region is crucial for maintaining not only CTCF loop anchors but also TADs.

To ascertain the integrity of cell-specific loops in the presence of the ΔZF1 mutant, we first identified cell-specific and ESC/NPC common (or constitutive) loops. As expected, the differentiation of WT ESCs into WT NPCs resulted in differential CTCF loop anchors (fig. S6A). Notably, the presence of CTCF and cohesin at DNA binding sites was mostly cell type invariant (fig. S6, B to D), and binding at cell-specific loop anchors was similar between the cell types (fig. S6, E to G). We then investigated whether cell-specific loop subsets were affected in ΔZF1. We found in ESCs that cell-specific CTCF anchors constituted 76% (1144/1504) of ΔZF1-lost anchors and 60% (1418/2356) of ΔZF1-retained anchors (Fig. 2D). Similarly, in NPCs, cell-specific CTCF anchors comprised 77% (1726/2238) of ΔZF1-lost anchors and 65% (1417/2257) of ΔZF1-retained anchors (Fig. 2D). Statistical analysis (Fisher’s exact test) indicated a significant difference in the distribution of cell-specific loops. The likelihood (odds ratio) of losing a cell-specific loop compared to losing a common loop was 2.1 times higher in ESCs and 1.8 times higher in NPCs. We show examples of NPC-specific, ΔZF1-lost loops at the *Podxl* (Fig. 2E) and *Grb10* (Fig. 2F) loci. These loci are illustrated as our subsequent studies will focus on the function of CTCF interaction with *Podxl* and *Grb10* RNAs. We highlight one of the loop boundaries at each locus, showing that the CTCF loop anchor coordinates are located within the *Podxl* (Fig. 2E) and *Grb10* (Fig. 2F) gene body. On the basis of these results, we concluded that the CTCF-ZF1 RNA binding region plays an essential role in maintaining loops not only in ESCs but also in the case of the reorganized CTCF loop anchors that arise after differentiation into NPCs.

### ΔZF1 loop loss is not due to loss of CTCF-DNA or CTCF-cohesin interactions

**Fig. 3.**
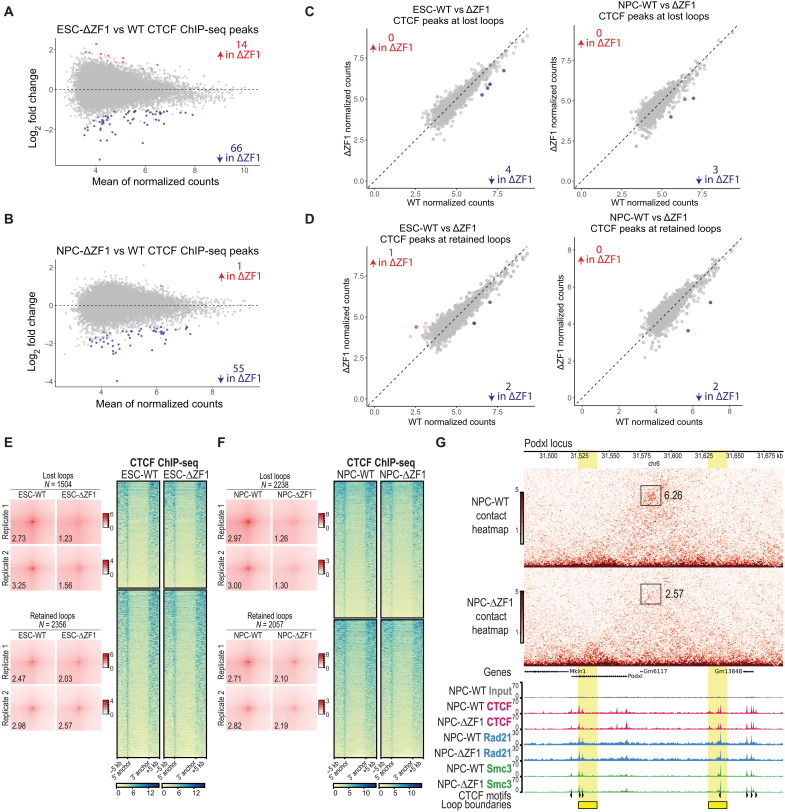
ΔZF1 loop loss is not due to changes in CTCF chromatin binding. (**A** and **B**) DiffBind MA plot (Deseq2 normalized) of differentially called peaks comparing ΔZF1 versus WT in (A) ESCs and (B) NPCs. Adjusted *P* value cutoff: ≤ 0.05; log_2_ fold change cutoff: ≥ 1, ≤ −1; *N* = 4 biological replicates. (**C** and **D**) Scatterplots showing DiffBind DESeq2-normalized counts of CTCF ChIP-seq peaks in WT and ΔZF1 conditions at loop anchors that are lost (C) or retained (D) in ΔZF1. (**E** and **F**) (Left) The APA plots per replicate (*N* = 2) in (E) ESCs and (F) NPCs show the comparison between WT and ΔZF1 for the lost and retained loop subsets. Numbers indicate APA scores. (Right) CTCF ChIP-seq heatmaps comparing CTCF chromatin binding in (E) ESC-WT and ESC-ΔZF1 and (F) NPC-WT and NPC-ΔZF1. Each row is a loop anchor coordinate, and the heatmap is clustered on the basis of whether the anchor is ΔZF1-lost (top cluster) or ΔZF1-retained (bottom cluster). (**G**) Micro-C contact heatmap (top) and ChIP-seq tracks for CTCF and cohesin subunits, Rad21 and Smc3 (bottom), at a representative *Podxl* locus. Loop boundaries are highlighted in yellow. Boxes on the contact heatmaps mark loop anchors. Numbers next to each box indicate the fold enrichment of pixel intensity at the corresponding anchor relative to the local background, as calculated with Cooltools. CTCF motifs with their orientation are annotated. The data in (E), (F), and (G) are represented as merged biological replicates (Micro-C *N* = 2; CTCF ChIP-seq *N* = 4; Rad21 and Smc3 ChIP-seq *N* = 2).

ΔZF1 has been shown to have an RNA binding defect, yet the loss of CTCF anchors may be due to other functional deficits arising from the deletion of ZF1. Therefore, we first probed whether the loss of CTCF anchors might arise from a decrease in CTCF DNA binding activity and/or changes in CTCF-protein interactions, particularly that of CTCF-cohesin. To address DNA binding activity, we performed CTCF ChIP-seq on WT and ΔZF1 rescues. Reproducible CTCF ChIP-seq peaks (present in three of four replicates) were identified using MACS2 (*50*), and differential peak calling was performed using DiffBind (*51*). We observed minimal changes in CTCF ChIP-seq peaks in the ΔZF1 mutant, with only 0.23% (66/28,455) of peaks significantly decreased in ESCs (Fig. 3A) and 0.24% (55/22,873) significantly decreased in NPCs (Fig. 3B). To specifically assess CTCF binding at loop anchors, we compared ChIP-seq signals between WT and ΔZF1 at anchors of lost and retained loops (Fig. 3, C and D, respectively). The vast majority of anchors maintained comparable CTCF occupancy in ΔZF1, with only three to four peaks at lost loops showing significantly reduced binding (log_2_ fold change ≤ −1; FDR ≤ 0.05). In accordance, ChIP-seq heatmaps of CTCF enrichment at ΔZF1-lost and ΔZF1-retained loop anchors revealed no notable differences between WT and ΔZF1 (Fig. 3, E and F).

We next investigated whether ΔZF1 could lead to a loss in CTCF-protein interactions. We performed native anti-Flag immunoprecipitation of Flag-Halo–tagged rescue CTCF from the chromatin fraction, followed by mass spectrometry (ChIP-MS) on WT and ΔZF1 rescues. As expected, we detected all the cohesin subunits as CTCF-protein interactors (fig. S7A and table S1). Moreover, we did not observe any differential protein interactions between ΔZF1 and WT (fig. S7A and table S1). This finding contrasts with that of another CTCF-ZF mutant (ΔZF10), with which a handful of differential interactions had been detected (fig. S7B and table S2). Consequently, we did not further investigate the ΔZF10 RNA binding mutant in this study as our focus was on the role of CTCF-RNA interactions. The intact CTCF-cohesin interactions in the ΔZF1 mutant observed by ChIP-MS is consistent with results from CTCF-IP and Rad21 immunoblots published previously (*31*).

Although a gross loss in CTCF-cohesin interactions was undetectable, we considered that there might be changes in CTCF-cohesin colocalization at CTCF anchors in the ΔZF1 mutant. Thus, we performed ChIP-seq of Rad21 and Smc3, two of the cohesin core subunits. There were minimal to no changes in Rad21 or Smc3 ChIP-seq peaks in ΔZF1 (fig. S7, C to F). We also found no differences in Rad21 or Smc3 ChIP-seq enrichment between WT and ΔZF1 at both ΔZF1-lost and ΔZF1-retained anchors (fig. S7, G to J). Figure 3G shows a representative *Podxl* locus wherein an NPC-specific chromatin loop is lost in NPC-ΔZF1, whereas CTCF, Rad21, and Smc3 DNA binding remain unchanged at the anchors. On the basis of these results, we concluded that the loss of CTCF loop anchors in the ΔZF1 mutant was not due to a loss of CTCF-DNA binding or CTCF-cohesin interactions and, instead, was most likely due to the loss of direct CTCF-RNA interactions.

### Dysregulated genes in the ΔZF1 mutant are enriched at disrupted loops

**Fig. 4.**
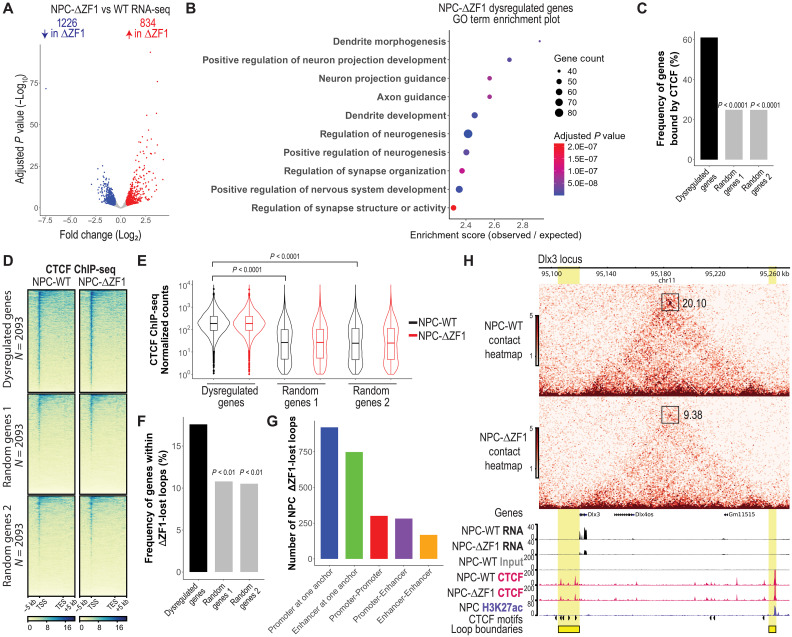
Dysregulated genes in NPC-ΔZF1 mutant are enriched at disrupted loops. (**A**) Deseq2 volcano plots showing gene expression changes in NPC-ΔZF1 compared to NPC-WT (see all in table S4). Adjusted *P* value cutoff: ≤ 0.05; log_2_ fold change cutoff: ≥ 0.5, ≤ −0.5; *N* = 2 biological replicates. (**B**) PANTHER GO term analysis of NPC-ΔZF1 dysregulated genes (see all in table S5). Shown are the top 10 terms related to neuronal function. (**C**) Bar plots showing the percentage of genes in each set (dysregulated, random 1, and random 2) that overlap with CTCF peaks in NPCs (−1 kb from the TSS to TES of genes). Statistical significance was determined using Fisher’s exact test. (**D**) NPC CTCF ChIP-seq heatmaps at dysregulated, random 1, and random 2 genes. The data are represented as merged biological replicates (*N* = 4). (**E**) Quantification of CTCF ChIP-seq reads at the TSS-TES of genes shown in (D). *P* values were determined using the Wilcoxon signed-rank test. (**F**) Bar plots showing the percentage of genes from each gene set (dysregulated, random 1, and random 2) that are colocalized within ΔZF1-lost loops in NPCs. *P* values were determined using Fisher’s exact test. (**G**) Bar plots showing the number of NPC-ΔZF1-lost anchors that overlapped with promoters and/or enhancers. (**H**) Example locus with decreased enhancer-promoter loop contact in ΔZF1. (Top) Micro-C contact heatmaps of WT compared to ΔZF1. CTCF anchors are highlighted in yellow. Boxes mark loop anchors; numbers next to them show fold enrichment over the local background, calculated with Cooltools. (Bottom) RNA-seq, CTCF, and H3K27ac ChIP-seq tracks. The upstream anchor is at the *Dlx3* promoter, and the downstream anchor is at a putative enhancer marked by H3K27ac (reanalyzed data; see table S6). The presence of CTCF motifs and their orientation is annotated. The data are represented as merged biological replicates (Micro-C *N* = 2; RNA-seq *N* = 2; CTCF ChIP-seq *N* = 4).

To assess the effect of the ΔZF1 mutant on gene expression in ESCs and NPCs, we performed bulk RNA-seq followed by DESeq2 analysis (*52*). We compared gene expression from ESC-ΔZF1 and NPC-ΔZF1 to their WT counterparts. In ESC-ΔZF1, we identified 432 dysregulated genes, with 235 down-regulated and 197 up-regulated (fig. S8A and table S3). In NPC-ΔZF1, we found 2060 dysregulated genes, with 1226 down-regulated and 834 up-regulated (Fig. 4A and table S4). Given that the change in gene expression was greater in the case of NPCs, we asked whether CTCF-ZF1 is crucial for an NPC-specific transcriptional program. We performed a Gene Ontology (GO) term enrichment analysis using PANTHER (*53*) and found that the dysregulated genes in NPC-ΔZF1 had an overrepresentation of terms related to NPC function, such as dendrite morphogenesis and development, regulation of neurogenesis, and nervous system development (Fig. 4B and table S5).

To assess whether gene expression changes were directly regulated by CTCF, we examined whether there was an enrichment of CTCF binding at dysregulated genes compared to randomly generated lists of genes. We found that 65% of ESC-ΔZF1 dysregulated genes (fig. S8B) and 61% of NPC-ΔZF1 dysregulated genes (Fig. 4C) were bound by CTCF, compared to 24 to 25% of random genes (Fig. 4C, S8B). CTCF ChIP-seq heatmaps revealed that dysregulated genes had a significant enrichment in CTCF binding, particularly at TSSs (Fig. 4D and fig. S8C). We quantified the CTCF ChIP-seq read counts across the genes [from the TSS to transcription end site (TES)] and found that CTCF binding was significantly more enriched at ΔZF1-dysregulated genes compared to random genes, with no changes in CTCF binding in the ΔZF1 mutant (Fig. 4E and fig. S8D).

We further investigated whether dysregulated genes were colocalized within ΔZF1-lost anchors. Our analysis revealed that 18% (80 of 436) of dysregulated genes in ESC-ΔZF1 (fig. S8E) and 18% (368 of 2093) of dysregulated genes in NPC-ΔZF1 were colocalized at disrupted CTCF anchors, compared to 10 to 11% of random genes (Fig. 4F). Statistical analysis indicates that dysregulated genes were significantly more likely to colocalize within ΔZF1-lost loops than would be expected by random chance (Fig. 4F and fig. S8E).

Next, we annotated the loop anchors on the basis of their overlap with promoters or putative enhancers. Putative enhancers were identified on the basis of ChIP-seq peaks of histone H3 acetylated at lysine-27 (H3K27ac) in ESCs and NPCs from previous studies (table S6) (*54*, *55*). We found several hundred ΔZF1-lost anchors overlapped with either a promoter (P) or enhancer (E) at one anchor or showed P-P, P-E, and E-E overlaps at both anchors (Fig. 4G and fig. S8F). We show an example of a ΔZF1-lost anchor wherein the upstream anchor overlaps with the promoter of a ΔZF1-down-regulated gene, *Dlx3* (Fig. 4H). In the ΔZF1 mutant, the CTCF-bound *Dlx3* promoter exhibited decreased looping contact with a downstream putative enhancer marked by H3K27ac, likely leading to decreased *Dlx3* expression (Fig. 4H).

Although some genes may be dysregulated indirectly due to a transcriptional cascade rather than direct chromatin loop changes, the higher enrichment of CTCF binding at dysregulated genes and the increased likelihood of dysregulated gene colocalization at ΔZF1-lost anchors suggest that disruptions in chromatin architecture directly contributed to transcriptional changes in the ΔZF1 mutant. These transcriptional changes can be partially explained by the disruption of CTCF-bound promoter-enhancer contacts.

### Identification of NPC-specific CTCF-ZF1 RNA interactions

**Fig. 5.**
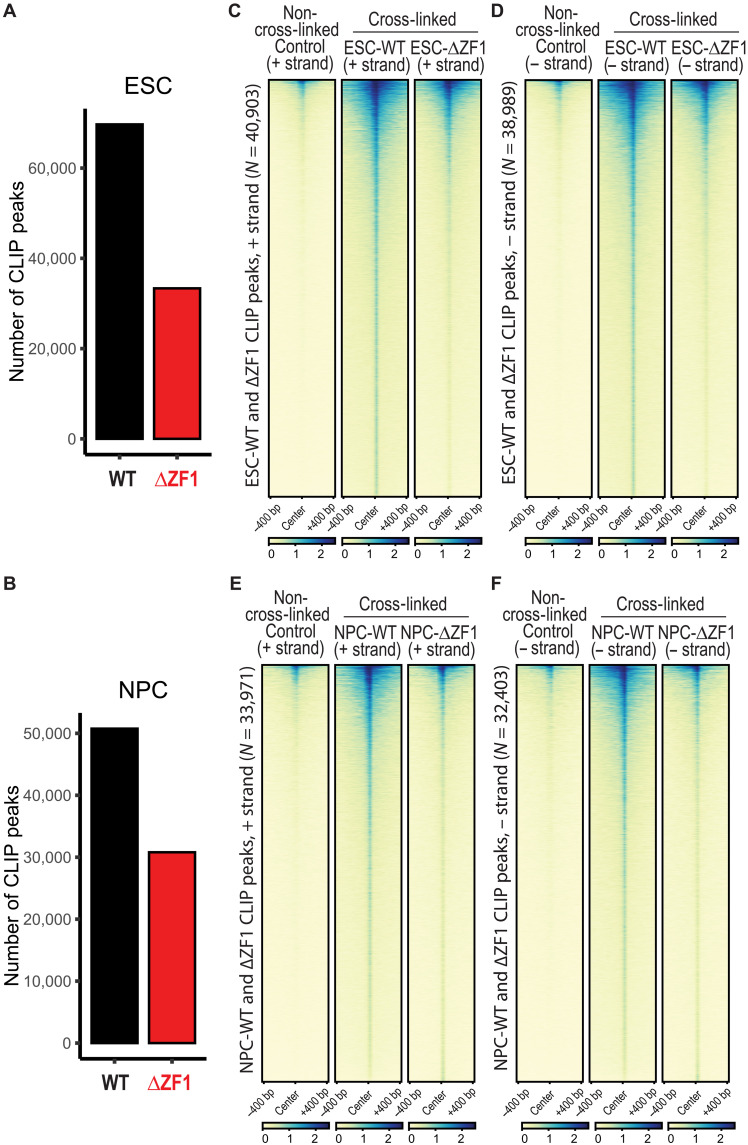
Decreased CTCF-RNA cross-linking in the ΔZF1 mutant. (**A** and **B**) ESCs (A) and NPCs (B) were UV cross-linked, and Flag-Halo–tagged WT or ΔZF1 rescue CTCF was immunoprecipitated using anti-Flag beads. RNA was purified, and the cDNA library was sequenced. Reproducible CLIP peaks were called (see Materials and Methods) in the WT and ΔZF1 mutant. Bar graphs of the number of reproducible peaks are shown (*N* = 3 to 4 biological replicates). (**C** and **D**) The combined set of CLIP peaks in WT and ΔZF1 was used to plot heatmaps of ESC-CTCF CLIP peak enrichment in the non-cross-linking negative control and cross-linked WT and ΔZF1. CLIP peaks are shown for the (C) plus (+) and (D) minus (−) strands. (**E** and **F**) Heatmaps of NPC-CTCF CLIP peak enrichment in the non-cross-linking negative control and cross-linked WT and ΔZF1. CLIP peaks are shown for the (E) plus (+) and (F) minus (−) strands.

In light of our findings thus far, we hypothesized that RNAs up-regulated in NPCs interact with CTCF to facilitate the formation of NPC-specific CTCF anchors in cis, i.e., at or near the site of transcription. To identify potential loop-inducing CTCF-RNA interactions, we performed iCLIP2 (crosslinking and immunoprecipitation followed by sequencing) (*56*, *57*). We used anti-Flag immunoprecipitation to purify Flag-Halo–tagged rescue CTCF (WT or ΔZF1) and verified CTCF-RNA interactions with RNA-^32^P labeling and CTCF immunoblotting (fig. S9A). We then sequenced the CTCF-RNA interactome in ESCs and NPCs. In ESCs, we identified 69,685 reproducible CLIP-seq peaks in CTCF-WT, whereas CTCF-ΔZF1 had 33,354 peaks (Fig. 5A). In NPCs, there were 50,758 peaks in CTCF-WT, whereas CTCF-ΔZF1 had 30,799 peaks (Fig. 5B). Most ΔZF1 peaks overlapped with WT peaks (fig. S9, B and C), consistent with an overall reduced RNA binding capacity in the mutant. CLIP-seq heatmaps of combined WT and ΔZF1 peaks confirmed a higher overall enrichment of CTCF-RNA cross-links in WT compared to ΔZF1 mutant in both ESCs (Fig. 5, C and D) and NPCs (Fig. 5, E and F). WT CLIP peaks were annotated to 6438 genes in ESCs and 6350 genes in NPCs. Consistently, CLIP-seq enrichment across the TSSs to TESs of annotated genes were decreased in ΔZF1 (fig. S9, D to G). CLIP peaks mapped to diverse RNA features—including 5′ untranslated regions (5′UTRs), 3′UTRs, exons, and introns—with a slight enrichment for intronic regions compared to background (non-cross-linked) peaks (fig. S10A). Gene annotations included coding, noncoding, antisense, and pseudogene transcripts, with coding RNAs representing the majority (fig. S10B). De novo motif analysis using MEME (*58*) identified significant RNA binding motifs, although they were present in only ~10 to 20% of peaks (fig. S10, C to E), suggesting possible recognition of RNA secondary structures rather than linear motifs.

**Fig. 6.**
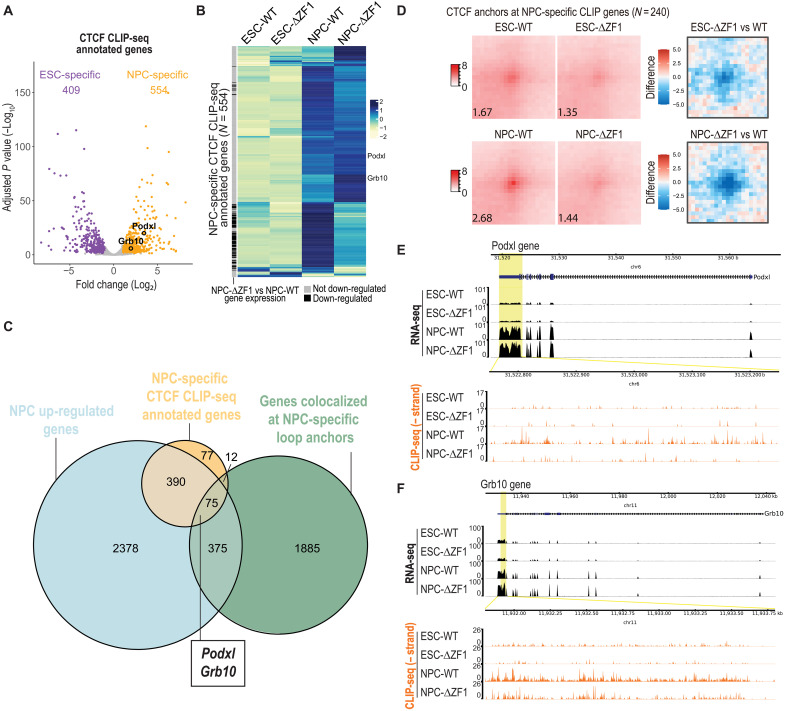
Identification of NPC-specific CTCF-RNA interactions. (**A**) Deseq2 volcano plot showing differential CTCF-RNA interactions comparing NPCs versus ESCs (see all in table S7). Each dot is a gene. Total CLIP-seq read counts from the TSS to TES of genes were compared. Adjusted *P* value cutoff: ≤ 0.05; log_2_ fold change cutoff: ≥ 1, ≤ −1; *N* = 3 to 4 biological replicates. (**B**) RNA-seq heatmaps of NPC-specific, CLIP-seq annotated genes, comparing ESC-WT, ESC-ΔZF1, NPC-WT, and NPC-ΔZF1. Each row is a gene and is annotated on the basis of whether gene expression in NPC-ΔZF1 is significantly down-regulated compared to WT. (**C**) Venn diagram of (i) genes up-regulated in NPCs relative to ESCs, (ii) genes colocalized at NPC-specific loop anchors, and (iii) genes annotated to be NPC-specific, CTCF-RNA interactors by CLIP-seq. Genes at the intersection of the three gene lists were considered for functional validation with *Podxl* and *Grb10* being selected. Both genes are annotated in (A) and (B). (**D**) (Left) APA plots at loop anchors that colocalized with RNAs at the intersection of three gene lists (C). (Right) Difference in APA enrichment between ΔZF1 and WT at these anchors. Data are represented as merged biological replicates (*N* = 2). (**E** and **F**) RNA-seq tracks (top) and CLIP-seq tracks (bottom) at genes of interest: (E) *Podxl* and (F) *Grb10*. CLIP-seq tracks are zoomed in for better visualization of nucleotide-level cross-links. The data are represented as merged biological replicates (RNA-seq *N* = 2; CLIP-seq *N* = 3 to 4).

To identify NPC-specific CTCF-RNA interactions, we used DESeq2 to call genes with significantly higher CLIP-seq reads in NPC WT compared to ESC WT. This analysis revealed 554 NPC-specific CLIP-seq genes (Fig. 6A and table S7). Expectedly, RNA-seq data showed that most of these genes were up-regulated in NPCs compared to ESCs (Fig. 6B). Although a subset of these NPC-specific genes showed reduced expression in the NPC-ΔZF1 mutant, most did not (Fig. 6B), indicating that the overall reduction in CTCF-RNA interactions observed in the mutant cannot be solely attributed to decreased RNA expression. To refine the list of RNA candidates for functional validation, we intersected three gene sets: (i) 554 NPC-specific CTCF-interacting genes identified by CLIP-seq, (ii) genes up-regulated in NPCs compared to ESCs, and (iii) genes that colocalize with NPC-specific CTCF loop anchors (Fig. 6C). This intersection yielded 75 RNAs that colocalized with 240 CTCF anchors. APA at these anchors revealed higher loop enrichment signal in NPC-WT compared to ESC-WT (Fig. 6D), with a more pronounced reduction in NPC-ΔZF1 than in ESC-ΔZF1 (Fig. 6D), suggesting that these loops exhibit increased RNA dependency in NPCs. From this subset, we selected *Podxl* and *Grb10* for further study on the basis of the following criteria: Their loci are associated with ΔZF1-lost, NPC-specific loops (Fig. 2, E and F), and they are expressed in the mouse brain and have established neuronal functions (*59*–*61*). Inspection of RNA-seq and CLIP-seq tracks confirmed their up-regulation in NPCs and revealed reduced CTCF-cross-linking interactions in NPC-ΔZF1, despite unchanged transcript levels (Fig. 6, E and F).

### Truncation of NPC-specific RNAs interacting with CTCF disrupts NPC-specific loops in cis

**Fig. 7.**
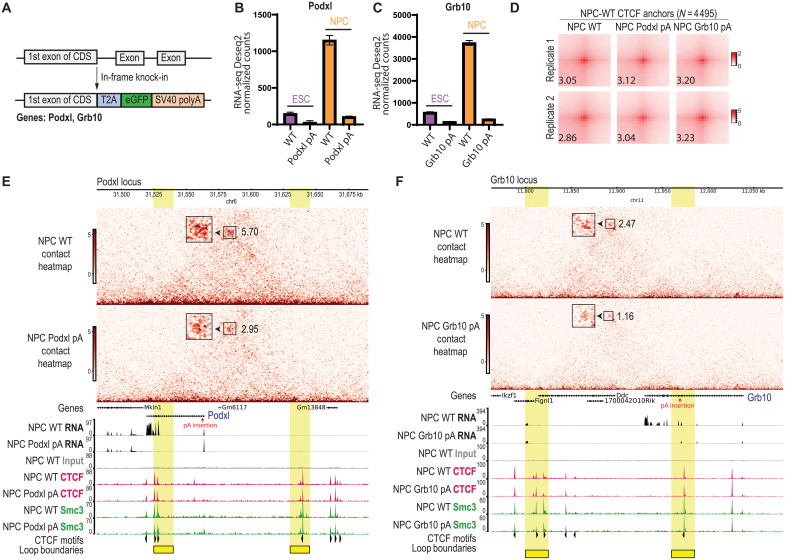
Truncation of NPC-specific RNAs interacting with CTCF leads to decreased chromatin loops in cis. (**A**) Schematic of the CRISPR-Cas9–mediated truncation of target RNAs, *Podxl* and *Grb10*. We generated a homozygous in-frame knock-in of T2A-eGFP-SV40pA downstream of the first exon of the coding sequence (CDS) of each gene. (**B** and **C**) RNA-seq Deseq2-normalized read counts of (B) *Podxl* in the WT and Podxl pA mutant and (C) *Grb10* in the WT and Grb10 pA mutant. Data are represented as means ± SEM, *N* = 3 biological replicates. (**D**) APA plots per replicate (*N* = 2) of NPC WT CTCF anchors, comparing WT, NPC Podxl pA, and NPC Grb10 pA. Numbers indicate APA scores. (**E** and **F**) Micro-C contact heatmaps (top) and RNA-seq tracks and ChIP-seq tracks (CTCF and Smc3) (bottom) at (E) the *Podxl* locus or (F) the *Grb10* locus. NPC WT is compared to either the Podxl pA mutant or the Grb10 pA mutant, respectively. Heatmaps were generated using ICE normalization implemented in cooler. Boxes on the contact heatmaps indicate loop anchors, enlarged for clarity. Numbers next to each box indicate the fold enrichment of pixel intensity at the corresponding anchor relative to the local background, as calculated with Cooltools. Loop boundaries are highlighted in yellow. The presence of CTCF motifs and their orientation is annotated. The data are represented as merged biological replicates (Micro-C *N* = 2; RNA-seq *N* = 2; CTCF and Smc3 ChIP-seq *N* = 2).

Transcriptional up-regulation of genes after differentiation into NPCs may facilitate chromatin looping through their RNA products interacting with CTCF at nearby CTCF binding sites. We hypothesized that truncation of the candidate RNAs, *Podxl* and *Grb10*, would decrease CTCF anchor enrichment in cis, specifically at the CTCF anchors where one anchor overlaps with the transcribed gene. To test this hypothesis, we disrupted the transcription of *Podxl* and *Grb10* RNAs independently by inserting T2A-eGFP followed by an SV40–polyA (pA) transcription termination signal downstream of the first exon of the coding sequence of each gene (Fig. 7A). Using CRISPR-Cas9 gene editing, we generated homozygous Podxl-T2A-eGFP-pA (Podxl pA) and Grb10-T2A-eGFP-pA (Grb10 pA) RNA truncation mutants (Fig. 7A). The truncation of *Podxl* and *Grb10* was verified by reverse transcription quantitative polymerase chain reaction (RT-qPCR) (fig. S11, A and B) and RNA-seq counts (Fig. 7, B and C). The mutant lines were eGFP fluorescent, resulting from in-frame knock-in of the repair template DNA (fig. S11C). Both RNA truncation mutants were able to differentiate into NPCs expressing the Sox1 marker (fig. S11D). The mutants did not show changes in CTCF or Rad21 protein levels (fig. S11E).

We performed Micro-C on the parental NPC WT cells, as well as NPC Podxl pA and Grb10 pA mutants, with higher read depth than previously used for the CTCF rescue lines (table S8) to resolve loops more clearly at the *Podxl* and *Grb10* loci. Replicate reproducibility was confirmed using HiCRep (*44*), which showed high stratum-adjusted correlation coefficients (SCC > 0.9) across all chromosomes (fig. S12A). Unlike the NPC-ΔZF1 mutant, we found that previously identified NPC-WT rescue CTCF anchors were, overall, not affected in either Podxl pA or Grb10 pA NPCs (Fig. 7D). However, CTCF anchor strength at the Podxl and Grb10 loci was significantly reduced in their respective pA mutants (Fig. 7, E and F), although the reduction was less pronounced than in NPC-ΔZF1. This reduction was also observed using an alternative normalization method (fig. S12, B and C) and was not seen at unrelated loci (fig. S12, D and E). The more modest reduction in pA mutants compared to ΔZF1 may reflect residual function of RNA fragments transcribed upstream of the polyadenylation site, which could partially preserve RNA-mediated loop stabilization. Notably, CTCF and Smc3 occupancy at these loci did not change significantly (Fig. 7, E and F), as confirmed by DiffBind quantification of ChIP-seq signal at loop anchors (fig. S12, F and G). The broader loop disruption observed in NPC-ΔZF1 compared to individual RNA truncations suggests that multiple RNAs cooperate with CTCF-ZF1 to maintain chromatin loops genome-wide.

In conclusion, the RNA binding region of CTCF, specifically the ZF1 region, is critical for maintaining NPC-specific chromatin loops at the *Podxl* and *Grb10* locus, among others. CTCF-ZF1 interacts with NPC-up-regulated RNAs, such as *Podxl* and *Grb10*. Disruption of CTCF-RNA interactions through either the deletion of ZF1 or the truncation of *Podxl* or *Grb10* RNA led to decreased chromatin loop enrichment in cis.

## DISCUSSION

CTCF is a key regulator of chromatin loops, but the mechanisms underlying how CTCF facilitates chromatin loops with cell type specificity have remained unclear. Our data suggest that cell type–specific RNAs orchestrate a subset of chromatin loops in cis through interactions with CTCF, adding nuance to our understanding of chromatin architecture during differentiation. The implications of our findings extend beyond the differentiation of ESCs into NPCs as subsets of chromatin loops that rely on specific CTCF-RNA interactions are likely relevant in other differentiation pathways. For example, CTCF is crucial in forming chromatin loops that drive myogenic transdifferentiation (*62*), cardiomyocyte development (*63*), and pancreatic cell differentiation (*64*). Furthermore, CTCF-RNA–mediated loops may provide a positive feedback mechanism for transcription. In this scenario, cell type–specific transcription factors initiate gene up-regulation, leading to CTCF-RNA–mediated promoter-enhancer looping, which, in turn, reinforces gene up-regulation.

The precise mechanisms by which CTCF-ZF1 RNA interactions facilitate chromatin loop formation during differentiation remain to be elucidated. Although we found some reduction in the levels of CTCF bound to its cognate sites in the ΔZF1 mutant, this reduction alone is insufficient to explain the far more pronounced loss of chromatin loops observed. Thus, the loss of CTCF anchors in ΔZF1 is not due to the loss of CTCF chromatin recruitment. In accordance, the truncation of CTCF-interacting RNAs, *Podxl* and *Grb10*, did not lead to decreased CTCF chromatin binding, despite an observable decrease in chromatin looping. Thus, the mechanism fostering CTCF loop anchor formation by ZF1-mediated RNA interaction appears distinctive from CTCF-mediated loop formation involving other, previously described noncoding RNAs (ncRNAs) and enhancer RNAs (eRNAs) given their primary role in either recruiting or releasing CTCF from chromatin. For example, long noncoding RNA (lncRNA), *Jpx*, binds to and extricates CTCF from low-affinity binding sites (*37*). Consequently, the loss of *Jpx* in ESCs leads to increased CTCF DNA binding and chromatin looping (*37*). Conversely, ncRNA *MYCNOS* was shown to be important in recruiting CTCF to the MYCN promoter in neuroblastoma cell lines (*38*), whereas eRNAs recruit CTCF to the boundary of the INK4a/ARF TAD in HeLa cells (*39*). Similarly, ncRNAs *Tsix* and *Xite* recruit CTCF to the X inactivation center (*35*). Whether the regulation of CTCF chromatin binding by these RNAs relies on known CTCF RNA binding regions (ZF1, ZF10, and/or RBRi) has not been investigated.

Although the truncation of *Podxl* and *Grb10* RNAs would also lead to a loss of their protein products, Podxl and Grb10 are mainly plasma membrane (*61*) and cytoplasmic (*65*) proteins, respectively, and do not directly interact with CTCF, based on CTCF ChIP-MS results (table S1). Hence, it is highly likely that the RNA molecules themselves, rather than their protein counterparts, are crucial for the direct maintenance of chromatin loops. Our study uncovered coding RNAs as being the predominant RNA biotype in our CTCF CLIP-seq. This makes the regulatory landscape of the genome more complex than previously envisioned, with coding RNAs playing multifaceted roles that extend beyond their traditional functions in certain genomic contexts. Future studies could explore how widespread this phenomenon is and whether other coding RNAs have similar structural roles in different cellular contexts.

Our study also suggests that the loss of CTCF loop anchors in the ΔZF1 mutant is not due to a loss of cohesin interaction or CTCF-cohesin colocalization on chromatin. This was an unexpected finding given the complete loss of chromatin loops upon cohesin degradation (*17*). It is possible that loop extrusion by cohesin initially brings CTCF anchors together and that CTCF-RNA interactions maintain the loop even after cohesin is unloaded, thus increasing loop lifetime. Therefore, CTCF-RNA interactions can independently contribute to chromatin loop maintenance, in addition to the well-established role of cohesin in loop extrusion. The possibility that RNA functions to block cohesin extrusion is not supported by cohesin ChIP-seq data because cohesin binding is not significantly decreased in either ∆ZF1 or RNA truncation mutants. Nonetheless, more technically refined studies that directly examine cohesin dynamics in the ∆ZF1 mutant may be informative for further elucidating the mechanistic role of RNA in loop regulation. On the basis of our results, it is likely that RNA contributes to both the formation and stabilization of a subset of loops by facilitating CTCF dimers or higher-order oligomers, as initially suggested in previous studies (*31*–*33*). Alternatively, RNA molecules may act as scaffolds or bridges that bring distant CTCF-bound chromatin regions into proximity, thereby promoting loop formation.

An intriguing and important aspect of our findings is that the CTCF-bound loops that were lost upon RNA truncation are in close proximity to the site of transcription. This putative cis-acting mechanism could be crucial in maintaining the integrity of chromatin loops in a localized or specific manner. The dual requirement of transcription and CTCF proximity could create an efficient system for dynamically regulating chromatin loops in response to cellular signals and differentiation cues. Future studies to explore how RNA proximity influences CTCF-mediated loop formation could entail artificial tethering of RNAs to distant sites or altering the site of transcription to assess its impact on loop stability.

The RNA motifs or secondary structures that mediate CTCF-RNA interactions are of substantial interest. Previous CTCF-RNA EMSAs (electrophoretic mobility shift assays) demonstrated that CTCF exhibits higher affinity for certain RNAs over others (*35*), suggesting specificity in these interactions. Our study supports this notion as not all NPC-up-regulated RNAs interacted with CTCF, indicating selective binding. In the absence of a prevalent RNA recognition motif, specificity is likely driven by RNA secondary or tertiary structures recognized by the ZF1 region. Notably, although the loss of CTCF anchors in the ΔZF1 mutant is likely attributable to disrupted RNA binding, ZF1-dependent loops are not significantly enriched for transcriptional activity or active histone marks. This observation highlights the need to further define the context-specific features that distinguish RNA-dependent CTCF anchors. Elucidating the structural and biochemical mechanisms by which CTCF cooperates with RNA to stabilize chromatin loops remains an important area for future investigation.

We acknowledge ongoing debate regarding the validity of CTCF-RNA interactions. A recent work from the Guttman lab (*66*), which introduced a denaturing CLAP protocol, concluded that some chromatin-associated proteins—including CTCF—may not directly bind RNA in vivo. Although we agree that distinguishing true biological interactions from experimental artifacts is essential, a subsequent reanalysis of these datasets (*67*) reaffirmed CTCF as a bona fide RNA binding protein, identifying RNA interactions consistent with earlier CLIP data. In parallel, independent orthogonal approaches further support the validity of CTCF-RNA interactions. RBR-ID has defined RNA binding regions within the CTCF zinc finger domain (*34*) and ChIRP-MS has detected CTCF as an RNA interactor (*68*). Collectively, these findings—including results from this study—suggest that CTCF-RNA interactions likely occur in vivo and serve biologically meaningful functions. Future applications of CLAP and related methods will help further clarify specificity and mechanistic roles.

Last, future explorations of the mechanisms by which CTCF-RNA interactions contribute to chromatin architecture may be impactful in the context of neuronal pathologies. CTCF is crucial for NPC proliferation, differentiation, and survival (*69*), and CTCF mutations are linked to neurological disorders such as intellectual disability (*70*), autism spectrum disorders (*71*), and neurodegenerative diseases (*72*). Although RNA does not universally determine CTCF-mediated looping at all chromatin loops, it serves an additional layer of regulation that complements CTCF function. By facilitating the formation of a subset of NPC-specific chromatin loops, CTCF-RNA interactions ensure the proper regulation of gene expression necessary for NPC function and differentiation. Understanding these interactions in greater detail could provide new insights into the molecular basis of neuronal function, in both health and disease.

### Limitations of the study

Our study provides notable insight into the role of CTCF-RNA interactions in chromatin loop maintenance in the context of cellular differentiation. However, some limitations should be acknowledged. First, our findings are based on in vitro models of ESC to NPC differentiation, which may not fully recapitulate the complexity of in vivo differentiation processes. We also did not disentangle the functions of the RNA molecules from their protein products. Although truncation of *Podxl* and *Grb10* RNAs led to decreased chromatin loop enrichment, it remains unclear whether the observed effects are solely due to the loss of RNA interactions with CTCF or whether the absence of the corresponding proteins also played an indirect role. Although we have demonstrated that CTCF-RNA interactions are required for maintaining chromatin loops, we have not demonstrated that these interactions are sufficient to establish or maintain these loops. Last, although our study focused on two RNA candidates, future research may systematically identify and validate additional RNAs involved in CTCF-mediated chromatin architecture across different cell types or developmental stages.

## MATERIALS AND METHODS

### Cell culture and NPC differentiation

E14Tga2 (American Type Culture Collection, CRL-1821) mouse ESCs (E14) were cultured in N2B27 media [Advanced Dulbecco’s modified Eagle’s medium (DMEM)/F12:Neurobasal-A (1:1) medium (Thermo Fisher Scientific, 12634028 and 10888022, respectively), with 3% ESC-grade fetal bovine serum (v/v, Gemini 900-108), 1x N2 supplement (Thermo Fisher Scientific, 17502-048), 1x B27 supplement (Thermo Fisher Scientific, 17504-044), 2 mM l-glutamine (Sigma-Aldrich, G7513), 1x Non Essential Amino Acids (Millipore, TMS-001-C), penicillin-streptomycin (0.1 mg/ml; Sigma-Aldrich, P0781), and 0.1 mM β-mercaptoethanol (Thermo Fisher Scientific, 21985023)] supplemented with leukemia inhibitory factor (homemade), 3 μM CHIR99021 (Tocris, 4423) and 1 μM PD0325901 (Sigma-Aldrich, PZ0162). Cells were grown on 0.1% gelatin-coated (Millipore, ES-006-B) plates at 37°C and 5% CO_2_. All cell lines were routinely checked for mycoplasma contamination and, in all cases, were negative.

In vitro differentiation of ESCs to NPCs has been described previously (*40*). Briefly, plates were coated overnight at 37°C with laminin (10 μg/ml; R&D Systems, 3446-005-01) in 1x phosphate-buffered saline (PBS). ESCs were dissociated with TrypLE Express (Gibco, 12605-028). Cells were seeded on laminin-coated plates at a pretitrated density of ~15,000/cm^2^. Titration of seeded ESCs is highly recommended due to variation between labs and cell lines. Cells were differentiated for 2 days in N2B27 media. NPC differentiation was confirmed by immunostaining with Sox1 antibody (R&D Systems, AF3369).

For CTCF rescue experiments, E14 CTCF-AID2 degron and dox-inducible CTCF-WT or ZF1 mutant rescue cells were treated with 1 μM 5-Ph-IAA (BioAcademia, 30-003) and dox (1 μg/ml; Sigma-Aldrich, D5207) for 24 hours and then harvested for downstream applications.

### CRISPR-Cas9 genome editing

Single guide RNAs (sgRNAs) were designed using CRISPR design tools in Benchling (https://benchling.com). sgRNA oligos (see table S9) were cloned into SpCas9-expressing PX330, PX458, or PX459 vectors (Addgene, 42230, 48138, and 62988, respectively). Briefly, vectors were digested with BbsI-HF (NEB, R3539). Forward and reverse sgRNA oligos were annealed together and phosphorylated using T4 Polynucleotide Kinase (PNK) (NEB, M0201). A 1:100 dilution of the resulting phosphorylated oligo duplex was ligated to the BbsI-digested vector using T4 DNA ligase (NEB, M0202). Donor plasmids were generated as described in the “Generation of plasmids and cell lines” section below (see also table S9 for cloning details). Plasmids were transformed into XL10 Gold (Agilent, 50-125-094) or Stellar Competent Cells (Takara Bio, 636766) at 1:10 dilution. All plasmids were confirmed by Sanger sequencing, which was done by Azenta Life Sciences or Psomagen. The sequencing results were aligned to the reference DNA using SnapGene.

For all transfections, ESCs at 70 to 80% confluency in 6-well plates containing 1 ml of media were used. Cells were transfected with 1 to 2 μg of donor plasmid and 0.5 μg of sgRNA plasmid, using 6 μl of Lipofectamine 2000 (Thermo Fisher Scientific, 11668027) in 100 to 150 μl of Opti-MEM Medium (Thermo Fisher Scientific, 31985062). Cells were incubated in transfection mix for 1 to 2 days before undergoing antibiotic or fluorescent selection. Cells were then fluorescence-activated cell sorting (FACS) sorted into 96-well plates (one cell per well) using a BD FACSAria II cell sorter. Single-cell clones were expanded, and genomic DNA (gDNA) was obtained using a DirectPCR Lysis Reagent (Viagen Biotech, 102-T). Successful gene editing was confirmed by genotyping PCR (see table S9 for genotyping primers) and, in addition, Western blots or flow cytometry analysis when applicable. Mutant PCR products from genotyping were also confirmed by Sanger sequencing.

### Generation of plasmids and cell lines

#### Endogenous CTCF-ΔZF1 mutants

E14 ESCs were transfected with PX458-eBFP2-sgRNA_CTCF_ZF1 (Addgene, 233090) targeting CTCF ZF1 and single-stranded donor DNA (IDT). The homozygous mutant clones were confirmed by PCR and Sanger sequencing.

#### CTCF-AID2 degron and rescue lines

First, the endogenous CTCF locus was tagged with mAID-eGFP upstream of the N terminus. The KL060–PBS-mAID-EGFP CTCF donor plasmid (Addgene, 232926) was generated by Gibson cloning of a gBlock [mAID-eGFP flanked by CTCF homology arms (IDT)] into the pBlueScript II SK (+) vector (see table S9 for details). E14 ESCs were transfected with the donor plasmid and PX330-sgRNA006_CTCF sgRNA plasmid (Addgene, 232928). eGFP-positive cells were sorted. Homozygous mAID-eGFP-CTCF ESCs (clone C7) were confirmed by genotyping and Western blot analysis.

Next, clone C7 was modified by knocking in mutant OsTIR(F74G) into the constitutively active Rosa26 locus. A plasmid expressing WT OsTIR1 flanked by Rosa26 homology arms (Addgene, 86233) was modified to express OsTIR1(F74G) instead (see table S9 for details). Clone C7 was transfected with the KL063–pCAGGS-OsTIR1(F74G)-BpA-Frt-PGK-EM7-NeoR-bpA-Frt-Rosa26 donor plasmid (Addgene, 232929) and sgRNA plasmid targeting the Rosa26 locus (Addgene, 64216). Cells were selected using a neomycin-resistance gene on the donor plasmid. Heterozygous OsTIR(F74G) (clone C7-E10) was confirmed by genotyping.

Although not used in this study, an mCherry reporter was knocked in at the Hb9/Mnx1 locus (Hb9-T2A-mCherry) of the C7-E10 clone. Hb9 is a motor neuron–specific marker. Initially, a motor neuron differentiation system was intended for this study, but we switched to the NPC differentiation system due to its higher efficiency. Cells were transfected with a double-stranded donor DNA (T2A-mCherry flanked by Hb9 homology arms) and the PX458-BFP-sgRNA011_Hb9 sgRNA plasmid. Homozygous Hb9-T2A-mCherry was confirmed by genotyping. The Hb9 mCherry reporter was further verified by anti-Hb9 immunostaining and flow cytometry analysis.

Next, clone C7-E10 was modified by knocking in a dox-inducible, Flag-Halo–tagged WT or ∆ZF1 CTCF at the constitutively active Tigre locus. The KL094–pTRE3G-Flag-Halo-CTCF_WT-BGHpA-CAGGS-rtta3G-rbgpA-Frt-PGK-EM7-PuroR-bpA-Frt TIGRE donor (Addgene, 232930) and KL095–pTRE3G-Flag-Halo-CTCF_ZF1_mutant-BGHpA-CAGGS-rtta3G-rbgpA-Frt-PGK-EM7-PuroR-bpA-Frt TIGRE donor (Addgene, 232931) plasmids were generated by modifying an existing plasmid (Addgene, 156432) that contains a constitutively expressed rtta3G cassette and a dox-inducible CTCF, flanked by Tigre homology arms (see table S9 for details). The donor plasmid and sgRNA plasmid targeting the Tigre locus (Addgene 92144) were transfected into the C7-E10 clone. Cells with the dox-inducible knock-in were selected using a puromycin-resistance gene on the donor plasmid. Homozygous knock-in clones (clone C7-E10-D11 for WT and C7-E10-F6 for ∆ZF1) were confirmed by genotyping. After selection, neomycin- and puromycin-resistance cassettes were excised using a plasmid containing FLP recombinase (Addgene, 13793).

#### Podxl and Grb10 pA lines

The donor plasmids were generated by first cloning ~1000 bp of *Grb10* or *Podxl* gDNA homology arms from E14 lysates into the pBlueScript II SK (+) vector (Agilent, 212207) (see table S9 for details). Using Gibson cloning, T2A-eGFP-SV40pA was then inserted in the middle of the gDNA such that it was flanked by ~500 bp of homology arms (see table S9 for details). E14 ESCs were transfected with KL157–pBlueScript II SK-Grb10_HA-T2A-eGFP-SV40PolyA (Addgene, 232935) or KL158–pBlueScript II SK-T2A-eGFP-SV40pA Podxl donor (Addgene, 232936) plasmids and sgRNA plasmids targeting the corresponding gene [PX459-sgRNA048_Grb10 (Addgene, 232934) or PX459-sgRNA047_Podxl (Addgene, 232932)]. Cells were selected using a puromycin-resistance gene on the sgRNA plasmid. eGFP-positive cells were then sorted. The homozygous mutant clones were confirmed by PCR and Sanger sequencing.

### Immunostaining and flow cytometry

Cells were dissociated into single cells with TrypLE Express, washed with 1x PBS, and fixed with 4% paraformaldehyde for 10 min at room temperature (r.t.). Cells were washed twice with FACS buffer (4% fetal bovine serum and 2 mM EDTA in 1x PBS) and permeabilized in 0.5% saponin in FACS buffer for 10 min at r.t. Saponin (0.5%) in FACS buffer was used for antibody incubation. Cells were incubated with goat anti-Sox1 primary antibody (R&D Systems, AF3369) overnight at 4°C [1:200 dilution from antibody stock (500 μg/ml)]. After washing, cells were incubated with anti-goat IgG-AF647 secondary antibody (Invitrogen, A32849) (1:1000 dilution) for 1 hour at 4°C.

For HaloTag labeling, cells were incubated in 37°C with 10 nM HaloTag-JF646 (Promega, GA1120) in media for 30 min. Cells were washed three times with 1x PBS and further incubated in media at 37°C for 30 min. Cells were then dissociated with TrypLE express and analyzed by flow cytometry.

Immunostained and HaloTag-labeled cells were assayed with a BD LSRII or Bio-Rad ZE5 flow cytometer, and results were analyzed using FlowJo software.

### Whole-cell extract and Western blotting

A confluent 6-well plate was lysed with 200 μl cold radioimmunoprecipitation assay (RIPA) buffer [20 mM Tris (pH 7.5), 150 mM NaCl, 1% SDS, 5% sodium deoxycholate, and 1% NP-40] supplemented with aprotinin (1 μg/ml), pepstatin A (1 μg/ml), leupeptin (1 μg/ml), and 0.2 mM phenylmethylsulfonyl fluoride (PMSF) protease inhibitors. The cell lysate was briefly probe sonicated (40% amplitude, five strokes) and centrifuged at 15,000*g* at 4°C for 10 min. The supernatant was collected, and protein concentrations were quantified via bicinchonic acid (BCA) assay (Thermo Fisher Scientific, A55861). Proteins were denatured in 1x Laemmli SDS–polyacrylamide gel electrophoresis (PAGE) buffer [63 mM tris-HCl (pH 6.8), 10% glycerol, 2% SDS, 0.0005% bromophenol blue, and 0.1% β-mercaptoethanol] at 95°C for 5 min. Twenty micrograms of total proteins was separated using a 6 to 15% SDS-PAGE gel and transferred onto a polyvinylidene difluoride membrane. Membranes were blocked with 5% milk in Tris-buffered saline with Tween-20 (TBST) at r.t. for 30 min and incubated with primary antibody overnight at 4°C (see table S10 for list of antibodies). Membranes were washed three times with TBST and then incubated with horseradish peroxidase (HRP)–conjugated secondary antibodies (see table S10 for list of antibodies) for 1 hour at r.t. HRP enzyme activity was detected by enhanced chemiluminescence (Thermo Fisher Scientific, 32106) and exposure to film or by chemiluminescence detection using a ChemiDoc MP imaging system (Bio-Rad).

### Micro-C

Micro-C was done as described previously for mammalian cells (*42*). Briefly, cells in a confluent 15-cm tissue culture plate (~30 million cells) were dissociated with TrypLE Express and cross-linked with freshly made 3 mM ethylene glycol bis(succinimidyl succinate) (EGS) in PBS (5 ml per 5 million cells) and incubated at r.t. for 45 min. Cross-linking reaction was quenched by adding Tris (pH 7.5) to a final concentration of 0.75 M and incubated at r.t. for 5 min. EGS-cross-linked cells were washed twice with 0.5% bovine serum albumin (BSA) in PBS and subjected to the second cross-linking with 1% formaldehyde in PBS (5 ml per 5 million cells) and incubated at r.t. for 10 min. Cross-linking reaction was quenched again by adding Tris (pH 7.5) to a final concentration of 0.75 M and incubated at r.t. for 5 min. Cells were washed twice with 0.5% BSA in PBS and aliquoted into 5 million cells per 1.5-ml tube. Cell pellets were spun down, snap-frozen, and stored at −80°C.

Cross-linked cells (5 million in 1.5-ml tubes) were thawed on ice and permeabilized in cold Micro-C Buffer (MB) #1 [50 mM NaCl, 10 mM Tris (pH 7.5), 5 mM MgCl_2_, 1 mM CaCl_2_, 0.2% NP-40, and 1x protease inhibitor cocktail] for 20 min on ice. Chromatin from permeabilized cells was digested by adding micrococcal nuclease (MNase) (NEB, M0247SVIAL) at 1:100 dilution and incubated at 37°C for 10 min, shaking at 850 rpm. Dilution of MNase was pretitrated such that chromatin is digested efficiently into nucleosome-sized fragments. Titration is recommended due to enzyme lot variability. Enzyme activity was stopped by adding EGTA to a final concentration of 4 mM and incubated at 65°C for 10 min. MNase-digested chromatin was washed twice with ice-cold MB #2 [50 mM NaCl, 10 mM Tris (pH 7.5), and 10 mM MgCl_2_].

Digested chromatin fragments were then subjected to 3′-dephosphorylation and 5′-phosphorylation using T4 PNK (0.5 U/μl) in 1x NEBuffer 2.1 (NEB, B6002S) with 2 mM adenosine triphosphate (ATP) and 5 mM dithiothreitol (DTT) and incubated for 15 min at 37°C with interval mixing. 3′-end-chewing was done by adding a DNA Polymerase I Klenow Fragment (0.5 U/μl) and incubating for another 15 min in 37°C. Blunt-end reaction was triggered by adding biotin-dATP, biotin–dCTP (deoxycytidine triphosphate), dGTP (deoxyguanosine triphosphate), and dTTP (deoxythymidine triphosphate) to a final concentration of 66 μM and incubated at 25°C for 45 min. The reaction was stopped by adding a 30 mM EDTA final concentration and incubating at 65°C for 20 min. Chromatin was washed once with cold MB #3 [50 mM Tris (pH 7.5) and 10 mM MgCl_2_].

Chromatin fragments with biotin-dNTPs were then ligated using T4 DNA ligase (20 U/μl; NEB, M0202M) and incubated at r.t. for at least 2.5 hours with slow rotation. Unligated ends containing biotin-dNTPs were then removed by exonuclease III in 1x NEBuffer 1 (5 U/μl; NEB, B7001S) and incubated at 37°C for 15 min. Chromatin was subjected to reverse cross-linking with proteinase K (1 mg/ml; Sigma-Aldrich, 3115879001), 1% SDS, and RNase A (100 μg/ml; Thermo Fisher Scientific, EN0531) and incubated at 65°C overnight.

DNA was extracted by adding 1x volume phenol:chloroform:isoamyl alcohol (25:24:1) solution (Thermo Fisher Scientific, 15593049) and isolating the aqueous phase after centrifugation. DNA was purified from the aqueous phase using a PCR purification kit (Zymo Research, DCC-5 D4013) following the manufacturer’s instructions. Separation of monomers and dimers was done by running purified DNA on 3% TBE agarose gel. DNA (200 to 400 bp) was then extracted from the gel by a Zymo Gel DNA recovery kit (Zymo Research, D4008).

DNA with biotin-dNTPs was captured by Dynabeads MyOne Streptavidin C1 (Thermo Fisher Scientific, 65001). Standard library preparation protocol including end-repair, A-tailing, and adaptor ligation was performed on beads with enzymes from a NEBNext Ultra II DNA Library prep kit for Illumina (E7645S). NEBNext Multiplex Oligos for Illumina (Dual Index Primers Set 1, E7600S) were used for barcoding. The sequencing library was amplified by a Kapa HiFi PCR enzyme (KAPA, KK2601). Libraries were verified by High Sensitivity D1000 ScreenTape (Agilent, 5067-5584) using the 4200 TapeStation System (Agilent). Libraries were sequenced as 2 x 50-bp or 2 x 150-bp paired-end reads on the Illumina Novaseq 6000 platform or Novaseq X platform, ensuring sufficient read depth of combined replicates for analysis (see table S8 for the number of reads in each sample replicate).

### Chromatin immunoprecipitation sequencing

ChIP-seq experiments were performed as described previously (*73*). Briefly, cells in a confluent 15-cm tissue culture plate were washed once with PBS and fixed for 10 min with 1% formaldehyde in 15 ml of fixation buffer [10 mM Hepes (pH 7.5), 15 mM NaCl, 0.15 mM EDTA, and 0.075 mM EGTA in DMEM]. Formaldehyde was quenched with 125 mM glycine and incubated for 5 min at r.t. Cells were washed and scraped in PBS and transferred to 15-ml tubes. Nuclei were isolated using buffers supplemented with 1x protease inhibitor cocktail (Roche, 11873580001) in the following order: 5 ml of LB1 [20 mM Tris (pH 7.5), 10 mM NaCl, 1 mM EDTA, and 0.2% NP-40; 10 min on ice], 5 ml of LB2 [20 mM Tris (pH 7.5), 200 mM NaCl, 1 mM EDTA, and 0.5 mM EGTA; 10 min on ice], and 1.2 ml of LB3 [20 mM Tris (pH 7.5), 150 mM NaCl, 1 mM EDTA, 0.5 mM EGTA, 1% Triton X-100, 0.1% sodium deoxycholate, and 0.1% SDS]. Nuclei in LB3 were transferred to 15-ml Bioruptor Pico tubes (Diagenode, C30010017) containing 800 mg of sonication beads. Chromatin was fragmented in LB3 to an average size of 200 to 250 bp using a Bioruptor Pico (Diagenode). The insoluble material was pelleted by centrifuging at 15,000*g* for 10 min at 4°C, and the supernatant was kept. The supernatant containing sonicated chromatin were precleared with 25 μl of Protein G Dynabeads (Invitrogen, 10004D) and incubated, rotating at 4°C for 1 hour. For each reaction, 2 to 4 μg of an antibody (see table S10 for list of antibodies) was incubated with 10 μl of Protein G Dynabeads in LB3, rotating at 4°C for 3 hours. Antibody-bead complexes were added to 300 μg of chromatin and incubated, rotating at 4°C overnight. Thirty micrograms of chromatin was used as input control. Beads were then washed four times with RIPA buffer [50 mM Hepes (pH 7.5), 500 mM LiCl, 1 mM EDTA, 0.5% sodium deoxycholate, and 1% NP-40] and washed once with TE50 buffer [10 mM Tris (pH 8.0), 1 mM EDTA, and 50 mM NaCl]. The bead-bound chromatin was eluted in 125 μl of elution buffer [50 mM Tris (pH 8.0), 10 mM EDTA, and 1% SDS)] and incubated at 65°C for 1 hour. Eluted chromatin was decross-linked using proteinase K (0.6 mg/ml) and RNase A (0.1 mg/ml) and incubated overnight at 55°C, shaking at 1000 rpm. A 1x volume phenol:chloroform:isoamyl alcohol (25:24:1) solution was added to the elution, and the aqueous phase was isolated after centrifugation. DNA was purified from the aqueous phase using a PCR purification kit (Zymo Research, DCC-5 D4013) following the manufacturer’s instructions. Libraries were prepared using a NEBNext Ultra II DNA Library prep kit for Illumina (E7645S) following the manufacturer’s instructions but using ½ volume for all the reactions. NEBNext Multiplex Oligos for Illumina (Dual Index Primers Set 1, E7600S) were used for barcoding. Fragments of 200 to 700 bp were size selected using Agencourt AMPure XP beads. Libraries were quantified with the High Sensitivity D1000 ScreenTape and TapeStation System. Libraries were sequenced as 2 x 50-bp or 2 x 150-bp paired-end reads on the Illumina Novaseq X platform.

### Chromatin immunoprecipitation–mass spectrometry

Cells were trypsinized from tissue culture plates, washed with 1x PBS, and pelleted. Nuclei were extracted by adding HMSD buffer [20 mM Hepes (pH 7.5) at 4°C, 5 mM MgCl_2_, sucrose (85.5 g/liter), 25 mM NaCl, and 1 mM DTT] supplemented with protease inhibitors [0.2 mM PMSF, pepstatin A (1 mg/ml), leupeptin (1 mg/ml), and aprotinin (1 mg/ml)] and rotated in a cold room for 30 min. Nuclei were spun down. The resulting pellet was resuspended in Buffer A [10 mM Tris (pH 8.0), 1.5 mM MgCl_2_, 10 mM KCl, and 0.2% NP-40] supplemented with protease inhibitors. Nuclei were rotated in Buffer A in a cold room for 30 min. The nuclear pellet was spun down. One milliliter of benzonase buffer [20 mM Tris (pH 8.0), 100 mM NaCl, and 2 mM MgCl_2_] with 1 μl of Benzonase (5 U/μl stock) (Sigma-Aldrich, E8263) was added to the chromatin pellet and incubated in a cold room, rotating to digest overnight. The insoluble material was pelleted, and the supernatant was kept for anti-Flag immunoprecipitation of Flag-Halo–tagged CTCF rescues. Total protein was quantified with BCA assay, and 400 to 600 μg of proteins was used for immunoprecipitation for each sample replicate. Ten microliters of anti-Flag agarose beads (Sigma-Aldrich, A2220) was added to the supernatant and incubated, rotating in a cold room overnight. After washing the beads three times with benzonase buffer, Flag-Halo-CTCF was eluted with 10 μl of 1x Flag peptide (5 mg/ml stock) (Sigma-Aldrich, F3290) in 90 μl of BC100 buffer [40 mM Tris (pH 7.3), 100 mM NaCl, 5 mM MgCl_2_, and 5% glycerol] supplemented with protease inhibitors. A first elution was done overnight at 4°C, and a second elution done for 2 to 3 hours at 4°C. Both elutions were combined. Ten to 20 μl of pooled elution was separated by SDS-PAGE, using 6 to 15% SDS-PAGE gradient gels. Silver staining and Western blots were done to confirm the presence of the bait (CTCF), as well as a known protein interactor (Rad21). Protein elutions were sent to the Rutgers University Mass Spectrometry Facility for a standard liquid chromatography–tandem mass spectrometry (LC-MS/MS) procedure. The facility performed differential spectral count analysis as previously described (*74*), and results are reported in tables S1 and S2.

### Bulk RNA-seq

Cells from a confluent 6-well plate were dissociated with TrypLE Express and washed once with 1x PBS. RNA was purified using an RNeasy Plus Mini Kit (Qiagen, 74136). RNA integrity number > 7 was verified using Agilent RNA ScreenTape (Agilent, 5067-5579). Ribosomal RNA (rRNA) was depleted from 1 μg of the starting total RNA using a NEBNext rRNA Depletion Kit v2 (NEB, E7405) following the manufacturer’s instructions. cDNA was synthesized using a NEBNext Ultra II RNA First Strand Synthesis module (NEB, E7771) (with random hexamers) and NEBNext Ultra II Directional RNA Second Strand Synthesis module (NEB, E7550) following the manufacturer’s instructions. Libraries were prepared using a NEBNext Ultra II DNA Library prep kit for Illumina (E7645S) in conjunction with NEBNext Multiplex Oligos for Illumina (E7600S or E7335S) for barcoding. Libraries were purified with Agencourt AMPure XP beads. Libraries were quantified with the High Sensitivity D1000 ScreenTape and TapeStation System. Libraries were sequenced as 2 x 50-bp paired-end reads on the Illumina Novaseq 6000 platform or Novaseq X platform.

### Reverse transcription quantitative polymerase chain reaction

Cells from a confluent 6-well plate were dissociated with TrypLE Express and washed once with 1x PBS. RNA was purified using an RNeasy Plus Mini Kit (Qiagen, 74136). cDNA synthesis was done on 2 μg of RNA using the Superscript III Reverse Transcriptase (Invitrogen, 18080044) and Oligo(dT)_20_ primer (Thermo Fisher Scientific, 18418020) in 20 μl of total volume. cDNA was diluted with 380 μl of nuclease-free water. Four microliters of diluted cDNA was used for qPCR. RT-qPCRs were performed in triplicate using PowerUp SYBR Green Master Mix (Thermo Fisher Scientific, A25742) on a CFX384 Touch Real-Time PCR detection system (Bio-Rad). Glyceraldehyde-3-phosphate dehydrogenase (GAPDH)–normalized relative expression and statistical analyses were calculated using CFX Maestro Software 2.3. The primers used are listed in table S9.

### CLIP-seq

CLIP-seq was based on a previously described iCLIP2 protocol (*56*) with modifications. A confluent 15-cm tissue culture plate was washed once with 1x PBS. Ten milliliters of 1x PBS was added to the plate, and cells were ultraviolet (UV) cross-linked on a plate with opened lid, at 400 mJ/cm^2^ using SpectroLinker XL-1000 (254 nm). Two 15-cm plates were scraped and combined. Cells were pelleted, snap-frozen, and stored at −80°C. ±UV cell pellets were resuspended in 20 ml of HMSD [20 mM Hepes (pH 7.5), 5 mM MgCl_2_, 25 mM NaCl, sucrose (85.5 g/liter), and 1 mM DTT] supplemented with protease inhibitors [0.2 mM PMSF, pepstatin A (1 mg/ml), leupeptin (1 mg/ml), and aprotinin (1 mg/ml)] and rotated in a cold room for 25 min. After washing with 1x PBS, nuclei were resuspended in 1 ml of cold PBS-lysis buffer (1 mM MgCl_2_, 0.1 mM CaCl_2_, 0.5% sodium deoxycholate, and 0.5% NP-40 in 1x PBS) supplemented with 1x EDTA-free protease inhibitor cocktail (Sigma-Aldrich, 11873580001), Protector RNase inhibitor (40 U/ml; Sigma-Aldrich, 3335402001), and 1 mM DTT and rotated in a cold room for 25 min. Twenty microliters of Turbo DNase (Thermo Fisher Scientific, AM2238) was added, and lysates were incubated at 37°C for 30 min, shaking at 1100 rpm. Partial RNA digestion was done with a pretitrated volume of dilute RNase I (Thermo Fisher Scientific, AM2295), incubated for 3 min at 37°C, and immediately transferred to ice. Two microliters of SUPERaseIN (Thermo Fisher Scientific, AM2696) and 0.1% final concentration of SDS was added to stop RNA digestion. Lysates were spun down, and the supernatant was transferred to new 1.5-ml tubes. Flag-tagged CTCF from the supernatant was immunoprecipitated with 5 μl anti-Flag magnetic beads (MilliporeSigma, M8823), rotating in a cold room for 2 hours. After four washes with PBS high-salt wash buffer (1 M NaCl, 0.1% SDS, 0.5% sodium deoxycholate, and 1% NP-40 in 1x PBS) and then four washes with PBS low-salt wash buffer (150 mM NaCl, 0.1% SDS, 0.5% sodium deoxycholate, and 1% NP-40 in 1x PBS), beads were treated with Turbo DNase (0.1 U/μl) with 1x protease inhibitor cocktail, Roche Protector RNase inhibitor (0.4 U/μl), and SUPERaseIn RNase inhibitor in 1x DNase buffer (0.1 U/μl; Thermo Fisher Scientific, AM2239). Beads were incubated at 37°C for 30 min, shaking at 850 rpm. Subsequently, RNAs were either radiolabeled for visualization or purified for cDNA library preparation.

For radiolabeling, on-beads RNA was 5′ labeled with ATP, [γ-^32^P] (0.45 mCi/ml; PerkinElmer, BLU002Z250UC) using T4 PNK (NEB, M0201L) in the supplied PNK buffer, supplemented with Roche Protector RNase inhibitor (0.9 U/μl). Standard precautions and proper waste disposal were observed when working with radioactive ATP. CTCF was eluted twice from beads using 1x Flag peptide (5 mg/ml stock) (Sigma-Aldrich, F3290) diluted 1:10 in PK buffer [100 mM Tris (pH 8.0), 150 mM NaCl, 5 mM EDTA, and 0.1% SDS]. Proteins were denatured with 1x NuPAGE LDS sample buffer (Thermo Fisher Scientific, NP0007) and incubated at 95°C for 5 min. The radiolabeled protein-RNA complexes were separated by electrophoresis using a 6% Bis-Tris gel and transferred to a nitrocellulose membrane. The radioactive membrane was exposed to film overnight at −80°C. CTCF Western blots were done on the same membrane as described in the “Whole-cell extract and Western blotting” section above.

For cDNA preparation, on-beads RNA was dephosphorylated at the 3′ end using T4 PNK and ligated to a DNA oligo (L3-App) adapter (see table S9) using T4 RNA Ligase 1 (NEB, M0204L). CTCF was eluted twice from beads using 1x Flag peptide (5 mg/ml stock) (Sigma-Aldrich, F3290) diluted 1:10 in PK buffer [100 mM Tris (pH 8.0), 150 mM NaCl, 5 mM EDTA, and 0.1% SDS]. Elutions were pooled, and proteins were denatured and digested with proteinase K (4 mg/ml) incubated in 55°C for 20 min, shaking at 1200 rpm. A 7 M final concentration of urea was added and incubated at 55°C for another 20 min. RNA-DNA hybrids were extracted using phenol:chloroform:isoamyl alcohol, separating the aqueous phase using phase lock gel tubes (VWR, 10847-802). From the aqueous phase, RNA-DNA hybrids were purified by ethanol precipitation overnight at −20°C. The RNA-DNA pellet was washed with ethanol and resuspended in nuclease-free water. Reverse transcription (RT) was done using Superscript III Reverse Transcriptase (Invitrogen, 18080044). The primer used for RT (RToligo) (see table S9) was complementary to the DNA oligo (L3-App) previously ligated to RNAs. Despite extensive digestion by proteinase K of cross-linked protein-RNA complexes, a small polypeptide remains covalently linked to RNA, which prematurely terminates RT, thereby providing positional information of the cross-linked nucleotides in the resulting cDNA library. Synthesized cDNAs were extracted using Dynabeads MyONE Silane beads (Thermo Fisher Scientific, 37002D). A second adapter ligation, with barcodes (see table S9), was done on the 3′ end of cDNA using T4 RNA Ligase 1. Adapter-ligated cDNAs were extracted using Dynabeads MyONE Silane beads. The adapter ligations are designed in such a way that sequencing reads start at the position where the cDNA was truncated during RT. Adapters contained unique molecular identifiers (UMIs) for duplicate removal during analysis. A cDNA preamplification was done on beads for six cycles using 2x KAPA HiFI Hot Start Mix (KAPA KK2601). The primers used were P5Solexa_s and P3Solexa_s (see table S9). ProNex Size-Selective Purification System (Promega NG2001) was used to size select the preamplified cDNA and remove primer-dimers. A second PCR amplification was done for nine cycles using primers P5Solexa and P3Solexa (see table S9). Libraries were size selected (200 to 500 bp) with Agencourt AMPure XP beads. Libraries were quantified with the High Sensitivity D1000 ScreenTape and TapeStation System. Libraries were sequenced as 100-bp single-end reads on the Illumina Novaseq 6000 platform.

### Computational analysis

#### Micro-C analysis

Micro-C analysis was performed according to the pipeline described by Dovetail Genomics in https://micro-c.readthedocs.io/en/latest/index.html. Briefly, multiple pairs of fastq files (two biological replicates per condition) were aligned to the mm10 reference genome using BWA-MEM split alignment. Pairtools was used for the next steps: identifying valid ligation events (pairs), removing PCR duplicates, and generating and down-sampling the final pairs files. Pairs files were down-sampled such that all samples being compared together had an equal number of valid pairs. The down-sampled pairs files were used for downstream analyses. Cool and mcool files were generated using cooler. HiC contact matrices were generated using Juicer Tools. APA plots and scores were generated using Juicer Tools. Micro-C contact heatmaps were generated using HiContacts and pyGenomeTracks. Reproducibility between replicates was determined using HiCRep (*44*). Quantification of CTCF anchors of interest was done using Cooltools (*75*).

Compartments were characterized as described previously (*76*). HiC-Pro was used to build contact matrices and perform iterative correction and eigenvector decomposition (ICE) normalization. HiC-Pro was used to generate ICE-normalized matrixes for 5- and 10-kb resolutions and annotation files that indicated the genomic bins. TADs were identified using Arrowhead at 10-kb resolution with default options. GENOVA was used to visualize ATA. Insulation score bigwig files were generated from cool files using Cooltools. Deeptools was used to plot insulation score profiles at CTCF ChIP-seq peaks.

#### Chromatin loop analysis

Chromatin loop analysis of Micro-C data was performed as previously described (*77*). Fithic2 was used to determine the contact counts of each pairwise 5-kb bins and calculate the likelihood of a contact to be significantly higher than expected for its genomic distance (*q* value). Loops were classified between pairwise samples (e.g., WT versus ΔZF1 and ESC versus NPC) as “sample1”-specific, “sample2”-specific, or common. A loop was considered sample1-specific if it was found to be significant only in sample1 but not in sample2, when using a *q* value cutoff of 0.01. To add more stringency, a loop was considered to be significantly different only if the log_2_ fold change of the contact counts was also ≤−1 or ≥1.

#### RNA-seq analysis

RNA-seq fastq files were processed using the route “rna-star” and “rna-star-groups-dge” from the Slide-n-Seq (sns) pipeline: https://igordot.github.io/sns/. Processing steps include alignment to the mouse reference genome (mm10) using the STAR aligner with default parameters. Counts were obtained using featureCounts. Deeptools was used to generate bigwig files from the merged bam files of replicates for the same condition. Bigwig tracks were visualized using IGV and pyGenomeTracks. Downstream analysis, including normalization and differential expression analysis, was performed using DESeq2. Genes were categorized as differentially expressed if log_2_ fold change ≤ −0.5 or ≥ 0.5 and adjusted *P* value ≤ 0.05. Complete differential gene expression analysis is listed in tables S3 and S4. GO term enrichment analysis of differentially expressed genes was performed using PANTHER. Complete GO terms are listed in table S5.

#### ChIP-seq analysis

ChIP-seq analysis was done as previously described (*73*). Reads were aligned to the mouse reference genome mm10, using Bowtie2 with default parameters. Reads of quality score less than 30 were removed using samtools, and PCR duplicates were removed using picard. Regions in the mm10 genome blacklist was removed using bedtools to generate the final BAM files. The final BAM files for replicates of the same condition were merged using samtools. Bigwig files were generated from merged BAM files using deeptools with parameters: --binSize 50 --normalizeUsing RPKM --ignoreDuplicates --ignoreForNormalization chrX --extendReads 250. Bigwig files were visualized using IGV and pyGenomeTracks. Peaks were called using MACS2 with parameters -g mm -B -f BAMPE --keep-dup all -q 0.01. Using MACS2-called peaks as input, differential ChIP-seq peaks and consensus peaks were identified using DiffBind. A peak was considered significantly different between samples if log_2_ fold change ≤ −1 or ≥ 1 and adjusted *P* value ≤ 0.05. Heatmaps were generated using the functions computeMatrix followed by plotHeatmap from deepTools. Violin plots for the ChIP-seq signal were prepared using an in-house R code as follows: Regions of interest (i.e., TSS-TES of genes) are imported from a BED-file as GRanges object using the GenomicRanges package. The number of ChIP-seq reads overlapping each region is counted from the BAM files using the getCounts function from the chromVAR package. Counts are Deseq2 normalized, and the mean count for each region is calculated from all replicates of each sample. The resulting counts file was used as input to ggplot2 for violin plots.

#### CLIP-seq analysis

CLIP-seq analysis was performed using publicly available code that was described previously (*57*). Briefly, FastQC was used to assess the quality of sequencing reads. Flexbar was used to demultiplex reads on the basis of barcodes and trim adapters. The reads were then aligned to the mm10 reference genome using STAR. PCR duplicates were removed using UMIs with UMI-tools. As described in the “CLIP-seq” protocol (above), reads start at the position where the cDNA was truncated during RT, making the position upstream of the 5′ end of the read the “cross-linked nucleotide.” Bedtools was used to convert BAM files to BED files, shift the BED intervals by 1 bp in the 5′ direction, and extract the 5′ positions of the new intervals. These positions were then piled up into BEDGraph files, separately for each strand. BEDGraph files were converted to BigWig files using bedGraphToBigWig. Bigwig files were visualized using IGV and plotted with pyGenomeTracks. For peak calling, BAM files from replicates of the same condition were merged and used as input for PureCLIP, which outputs individual cross-link sites. PureCLIP-called cross-link sites were postprocessed as described previously (*57*) to obtain equal-sized CLIP peaks of 300 nucleotides. For each called peak, the number of cross-link events per replicate was determined. A minimum cutoff (10th percentile of the number of cross-linked sites) was calculated for each replicate. A peak was considered reproducible if it met the cutoff in a sufficient number of replicates (at least three of four replicates, except for NPC WT, where the criterion was two of three replicates). Peak calling was also performed for non-cross-linked negative controls, and peaks with at least 20% overlap with negative control peaks were filtered out. Reproducible CLIP peaks were assigned to gene names and gene types on the basis of GENCODE annotations (GRCm38.p4). De novo RNA motif analysis using the reproducible CLIP peaks as input was performed using MEME. The assignment of CLIP peaks to genomic features (e.g., promoters, introns, and exons) was done using ChIPseeker. Differential CLIP genes between ESCs and NPCs were identified by combining unique annotated CLIP genes from both cell types and retrieving the TSS-TES regions as a BED file. The BED file was imported as a GRanges object using GenomicRanges package on R, and the number of CLIP-seq reads overlapping each region was counted from the BAM files using the getCounts function from the chromVAR package. DESeq2 was used to normalize the read counts and identify differential genes (log_2_ fold change cutoff ≤ −1 or ≥ 1; adjusted *P* value ≤ 0.05). Heatmaps of CLIP peaks and CLIP-seq signal from TSS-TES regions of annotated genes were generated from bigwig files using deepTools. Venn diagrams were drawn using the VennDiagram package on R studio.

### Statistical analysis

Statistical analysis related to experiments have been described above in each section.
