# Supplementary material for: CTCF-RNA interactions orchestrate cell-specific chromatin loop organization
Source: Sci Adv. 2025 Nov 26;11(48):eady5507. doi: 10.1126/sciadv.ady5507 (PMC12652319; doi:10.1126/sciadv.ady5507)
Supplement: Supplementary file 1 — Figs. S1 to S12 Legends for tables S1 to S5, and S7 to S9 Tables S6 and S10 [file sciadv.ady5507_sm.pdf]

Science Advances
AAAS

Supplementary Materials for

CTCF-RNA interactions orchestrate cell-specific chromatin loop organization

Kimberly Lucero *et al.*

Corresponding author: Danny Reinberg, dxr1274@miami.edu

Sci. Adv. **11**, eady5507 (2025)
DOI: 10.1126/sciadv.ady5507

The PDF file includes:

Figs. S1 to S12
Legends for tables S1 to S5, and S7 to S9
Tables S6 and S10

Other Supplementary Material for this manuscript includes the following:

Tables S1 to S5, and S7 to S9

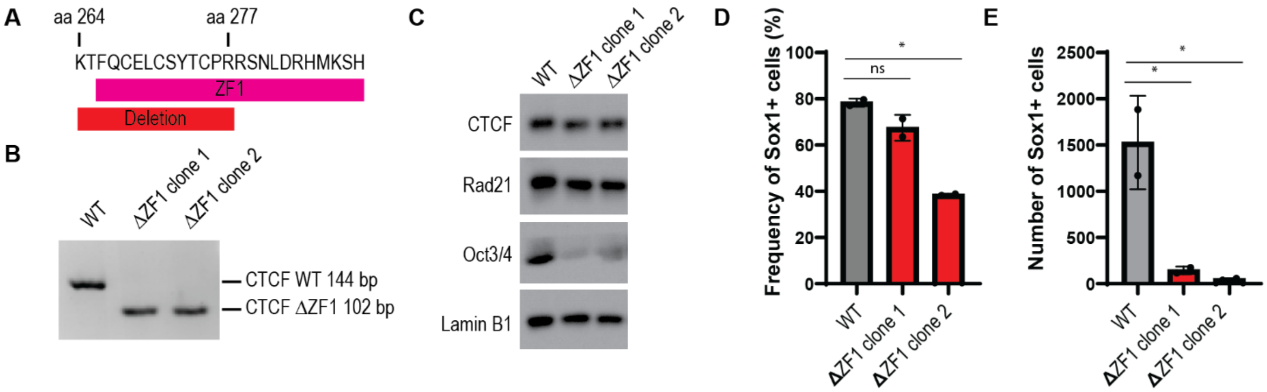

Fig. S1. Endogenous CTCF-ΔZF1 mutants do not efficiently differentiate into NPCs. (**A**) Schematic of 14-amino acid (aa) deletion of CTCF-ZF1 in ESCs. (**B**) Genotyping of endogenous CTCF-ΔZF1 clones. Deletion was confirmed by DNA sequencing of purified bands. (**C**) Western blot of parental WT ESCs and ΔZF1 clones. (**D**) Percentages of Sox1+ cells after 2 days of NPC differentiation. Cells were fixed and immunostained with anti-Sox1 antibody and analyzed by flow cytometry. Data are represented as mean ± SEM, p-values were determined using Dunnett's multiple comparison test, n.s.=not significant, * $p < 0.05$, N=2 biological replicates. (**E**) Number of Sox1+ cells per 5,000 seeded ESCs, after 2 days of NPC differentiation. Cells were fixed and immunostained with anti-Sox1 antibody and analyzed by flow cytometry. Cell numbers were normalized by cell counting beads. Data are represented as mean ± SEM, p-values were determined using Dunnett's multiple comparison test, * $p < 0.05$, N=2 biological replicates.

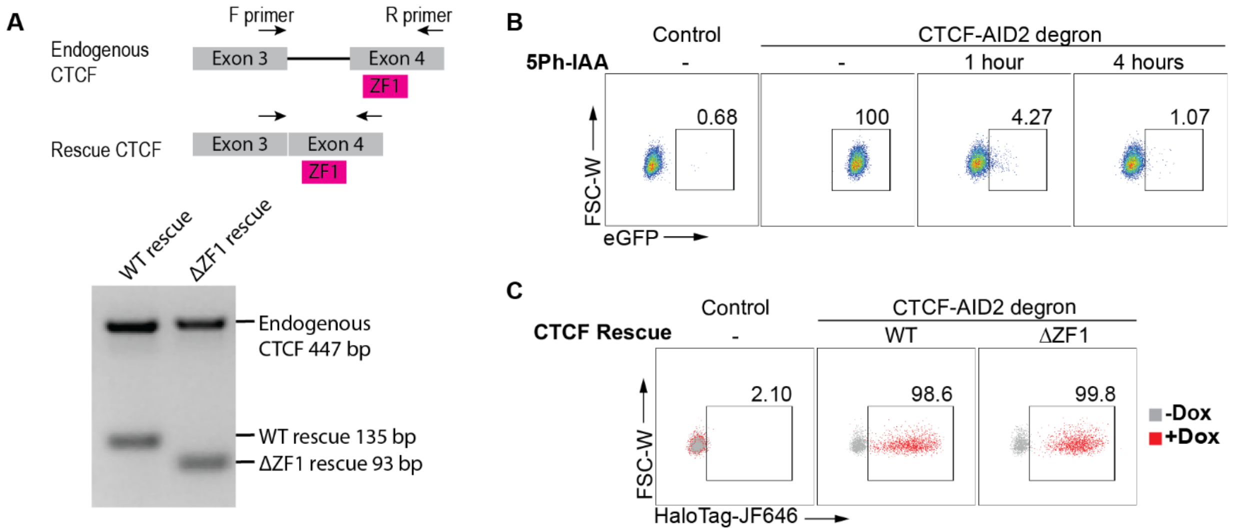

Fig. S2. Validation of CTCF-AID2 and rescue lines. (**A**) The schematic (above) shows the genotyping strategy with the arrows indicating forward (F) and reverse (R) primers. Primers amplify both endogenous and rescue CTCF but result in different DNA base pair (bp) lengths. Below is the genotyping of CTCF-AID2 and rescue lines. (**B**) CTCF-AID2 degron line after no treatment, 1 hr, or 4 hr of treatment with 5-Ph-IAA. eGFP fluorescence was then analyzed by flow cytometry. The negative control is the parental E14 WT cell line. Numbers are the percentages of cells within the boxed area among all cells in the plot. (**C**) CTCF-AID2 degron line after no treatment, or 24 hr of treatment with dox. Cells were labeled with HaloTag-JF646. JF646 fluorescence was then analyzed by flow cytometry. The negative control is the parental E14 WT cell line. Numbers are the percentages of cells within the boxed area among all dox-treated cells (in red) in the plot.

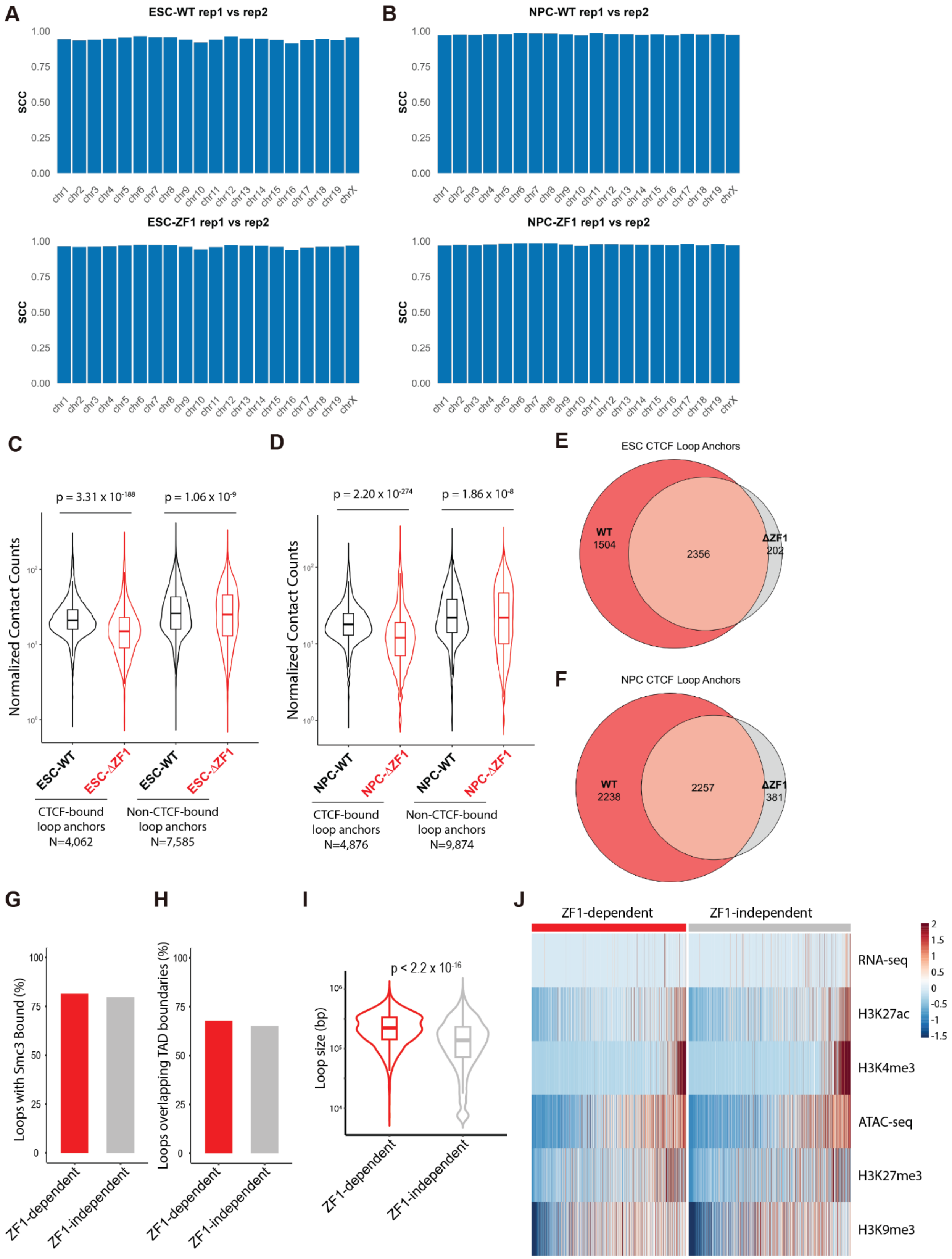

Fig. S3. Characterization of ZF1-dependent and ZF1-independent CTCF anchors. (**A, B**) HicRep was used to calculate stratum-adjusted correlation coefficients (SCC) between replicates in ESCs (A) and NPCs (B). Replicates of the same condition (WT or ΔZF1) have high reproducibility with SCC > 0.9. (**C, D**) Chromatin loops were identified

using FitHiC2 (5-kb resolution, FDR ≤ 0.05) and classified as CTCF-bound or non-CTCF-bound based on overlap with CTCF ChIP-seq peaks. Violin plots show FitHiC2 contact counts from merged biological replicates (N = 2) in ESCs (C) and NPCs (D). p-values were calculated using the Wilcoxon signed-rank test. (**E, F**) CTCF loop anchors were called independently for WT and ΔZF1, and differential loop anchors were identified (see Methods). Venn diagrams show the intersection of CTCF loop anchors in WT and ΔZF1 samples in ESCs (E) and NPCs (F). (**G**) ZF1-dependent (ΔZF1-lost) and ZF1-independent (ΔZF1-retained) loops in ESCs were overlapped with Smc3 (cohesin core subunit) ChIP-seq peaks. Bar plots show the percentage of loops bound by Smc3. (**H**) ZF1-dependent and ZF1-independent loops were overlapped with TAD boundaries in ESCs. Bar plots show the percentage of loops colocalized with TAD boundaries. (**I**) Distances between loop anchors were quantified for ZF1-dependent and ZF1-independent loops in ESCs. p-value was determined using the Wilcoxon signed-rank test. (**J**) RNA-seq (this study) and published datasets (H3K27ac, H3K4me3, ATAC-seq, H3K27me3, H3K9me3) in ESCs were used to quantify transcription, chromatin accessibility, and histone modifications at ZF1-dependent and ZF1-independent loop anchors. Heatmaps display z-score normalized signal at anchor sites.

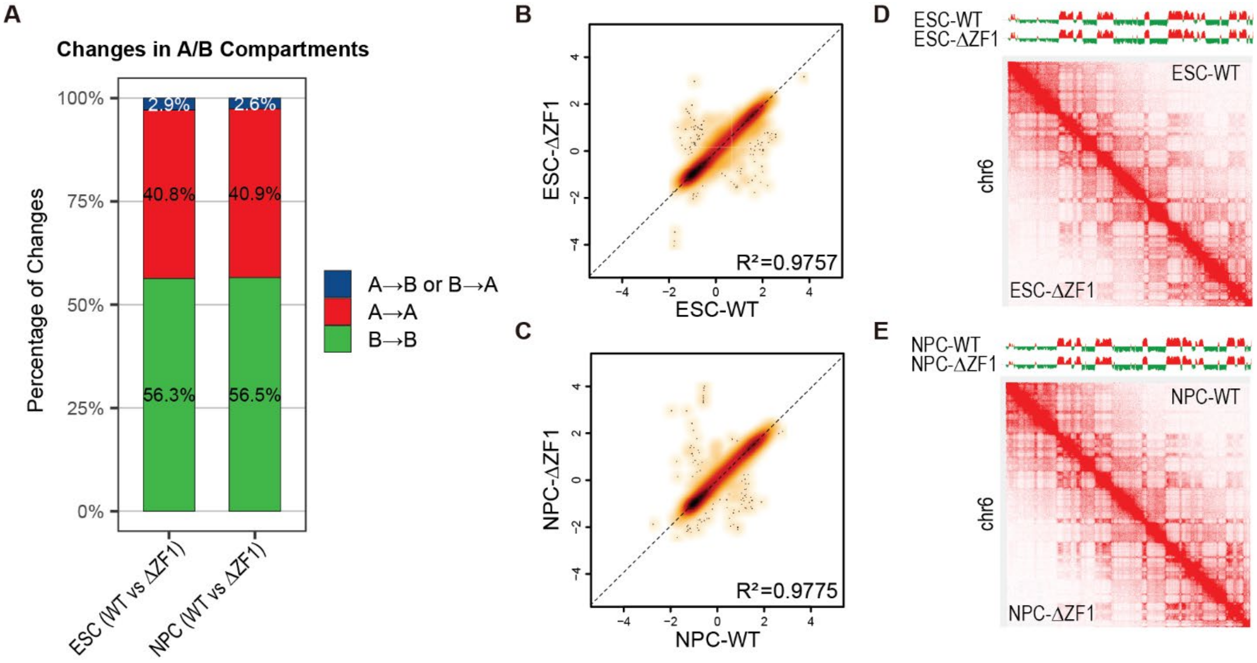

Fig. S4. Genomic compartmentalization is minimally changed in ΔZF1 for both ESCs and NPCs. (**A**) Compartments were analyzed at 10-kb resolution for both WT and ΔZF1. Bar graphs show the percentage of compartment changes from WT to ΔZF1. (**B**, **C**) Scatter plots comparing the first eigenvector values (equivalent to first principal component) between WT and ΔZF1 in (B) ESCs and (C) NPCs. The correlation coefficient (R^2) was calculated. Dashed lines represent linear regression lines. (**D**, **E**) The first eigenvector tracks (top) and Micro-C heatmaps (bottom) comparing WT and ΔZF1 in (D) ESCs and (E) NPCs. Data is represented as merged biological replicates (N=2).

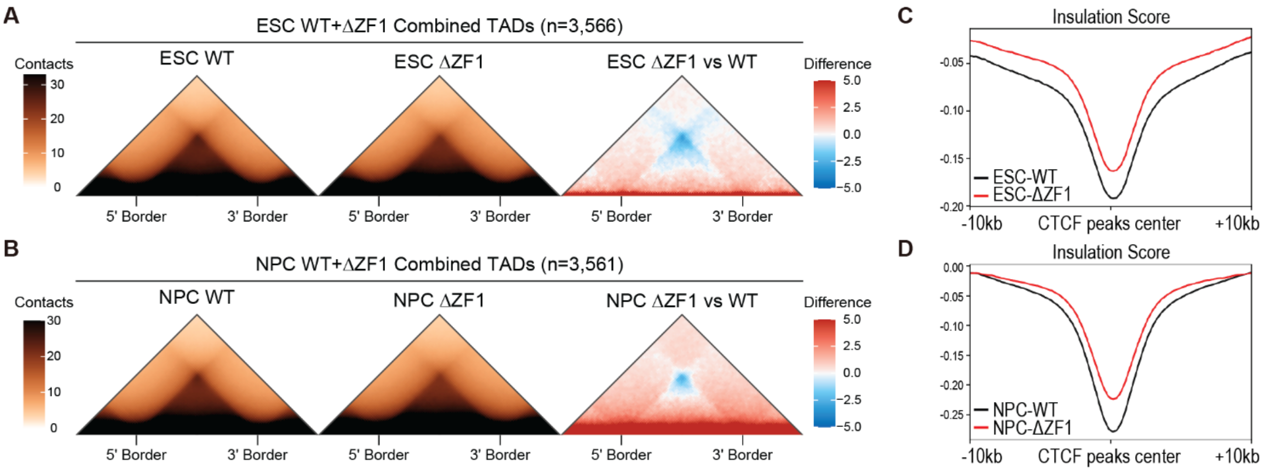

Fig. S5. TADs and TAD boundaries are weakened in ΔZF1 for both ESCs and NPCs. (**A**, **B**) Aggregate TAD analysis (ATA) of WT (left), ΔZF1 (middle), and the difference between ΔZF1 and WT (right) in (A) ESCs and (B) NPCs. TADs were called for both WT and ΔZF1 using Arrowhead at 10-kb resolution. The combined TADs in both conditions were used to plot ATA. (**C**, **D**) Diamond insulation scores (1kb resolution, 10kb window size) of WT and ΔZF1 centered at CTCF ChIP-seq peaks in (C) ESCs and (D) NPCs. Data is represented as merged biological replicates (N=2).

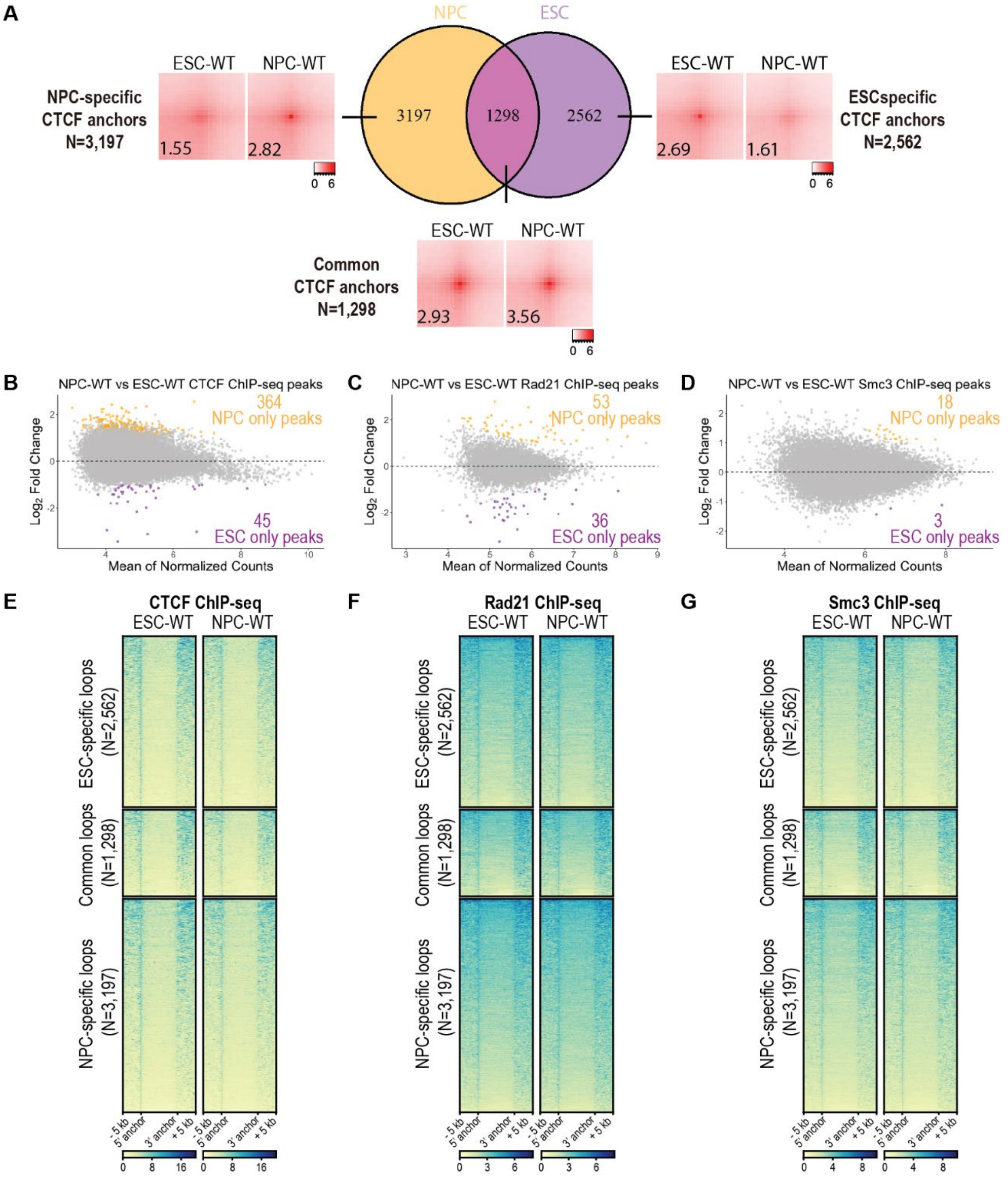

Fig. S6. ESCs and NPCs exhibit differential chromatin loops, but CTCF and cohesin binding are mostly cell-type-invariant. (**A**) Venn diagram of NPC and ESC CTCF anchors. APA plots show aggregated peaks from each of the NPC-specific, ESC/NPC common, and ESC-specific loop subsets. Numbers indicate APA scores. (**B**, **C**, **D**) DiffBind MA plot (Deseq2 normalized) of differentially called (B) CTCF, (C) Rad21, and (D) Smc3 ChIP-seq peaks between NPC-WT vs ESC-WT. Adjusted p-value cutoff: ≤ 0.05, $\log_2$ fold-change cutoff: ≥ 1, ≤ -1, biological replicates: CTCF N=4, Rad21 N=2, Smc3 N=2. (**E**, **F**, **G**) ChIP-seq heatmaps of (E) CTCF, (F) Rad21, and (G) Smc3, comparing chromatin binding in ESC-WT and NPC-WT. Each row is a loop anchor coordinate, and the heatmap is clustered based on whether the anchors are ESC-specific, ESC/NPC common, or NPC-specific. Data is represented as merged biological replicates (CTCF N=4, Rad21 N=2, Smc3 N=2).

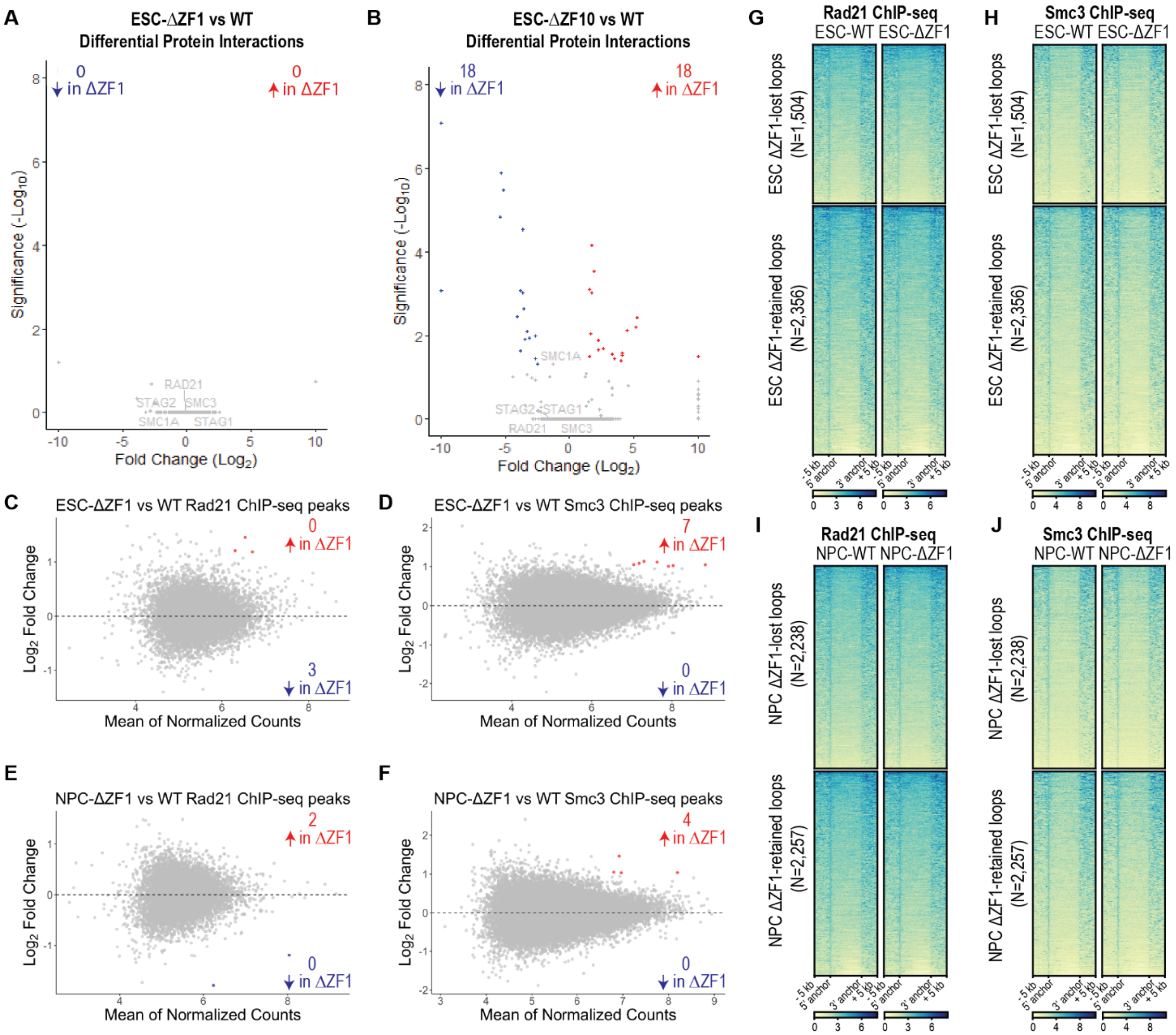

Fig. S7. CTCF-cohesin interactions and chromatin colocalization are not disrupted in ΔZF1 mutants. (**A**, **B**) Volcano plot of CTCF differential protein interactions comparing ESC WT vs (A) ESC-ΔZF1 or (B) ESC-ΔZF10 (see all in **Table S1** or **Table S2**, respectively). Flag-Halo-tagged CTCF was purified by anti-Flag immunoprecipitation in native conditions and protein interactions were identified by MS. Adjusted p-value cutoff: ≤ 0.05, $\log_2$ fold-change cutoff: ≥ 1, ≤ -1, N=2 biological replicates. (**C**, **D**) DiffBind MA plot (Deseq2 normalized) of differentially called (C) Rad21 and (D) Smc3 ChIP-seq peaks comparing ESC-ΔZF1 vs WT. Adjusted p-value cutoff: ≤ 0.05, $\log_2$ fold-change cutoff: ≥ 1, ≤ -1, N=2 biological replicates. (**E**, **F**) DiffBind MA plot of differentially called (E) Rad21 and (F) Smc3 ChIP-seq peaks comparing NPC-ΔZF1 vs WT. Adjusted p-value cutoff: ≤ 0.05, $\log_2$ fold-change cutoff: ≥ 1, ≤ -1, N=2 biological replicates. (**G**, **H**) ChIP-seq heatmaps of (G) Rad21 and (H) Smc3, comparing chromatin binding in ESC-ΔZF1 vs WT. Each row is a loop anchor coordinate, and the heatmap is clustered based on whether the anchors are ΔZF1-lost or ΔZF1-retained. (**I**, **J**) ChIP-seq heatmaps of (I) Rad21 and (J) Smc3, comparing chromatin binding in NPC-ΔZF1 vs WT. Each row is a loop anchor coordinate, and the heatmap is clustered based on whether the anchors are ΔZF1-lost or ΔZF1-retained. Data is represented as merged biological replicates.

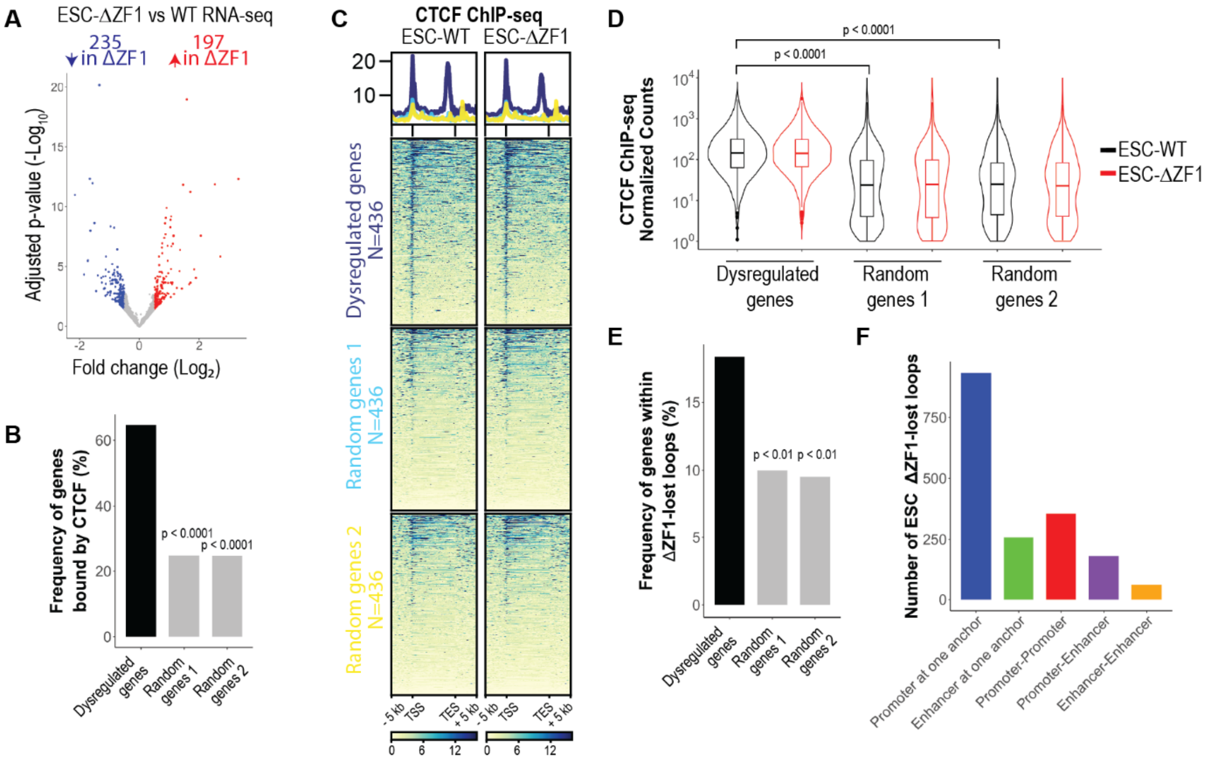

Fig. S8. Dysregulated genes in ESC-ΔZF1 mutant are enriched at disrupted loops. (**A**) Deseq2 volcano plots showing gene expression changes in ESC-ΔZF1 compared to ESC-WT (see all in **Table S3**). Adjusted p-value cutoff: ≤ 0.05, $\log_2$ fold-change cutoff: ≥ 0.5, ≤ -0.5, N=2 biological replicates. (**B**) Bar plots showing the percentage of genes from each gene set (dysregulated, random genes 1, random genes 2) that overlap with CTCF ChIP-seq peaks in ESCs. Overlaps were defined as CTCF peaks located within 1 kb upstream of the transcription start site (TSS) to the transcription end site (TES). Statistical significance between dysregulated genes and each random gene set was assessed using Fisher's exact test. (**C**) CTCF ChIP-seq heatmaps at the TSS to TES of dysregulated genes and two sets of randomly generated genes in ESCs. (**D**) Violin plots quantifying CTCF ChIP-seq reads from TSS to TES of genes shown in (C). Counts are represented as the average of 4 biological replicates per condition. p-values were determined using Wilcoxon signed-rank test. (**E**) Bar plots showing the percentage of genes from each gene set (dysregulated, random genes 1, random genes 2) that are co-localized within ΔZF1-lost loops in ESCs. p-values comparing each of the random gene sets to dysregulated genes were determined using Fisher's Exact test. (**F**) Bar plots showing the number of ESC-ΔZF1-lost anchors that overlapped with promoters and/or enhancers.

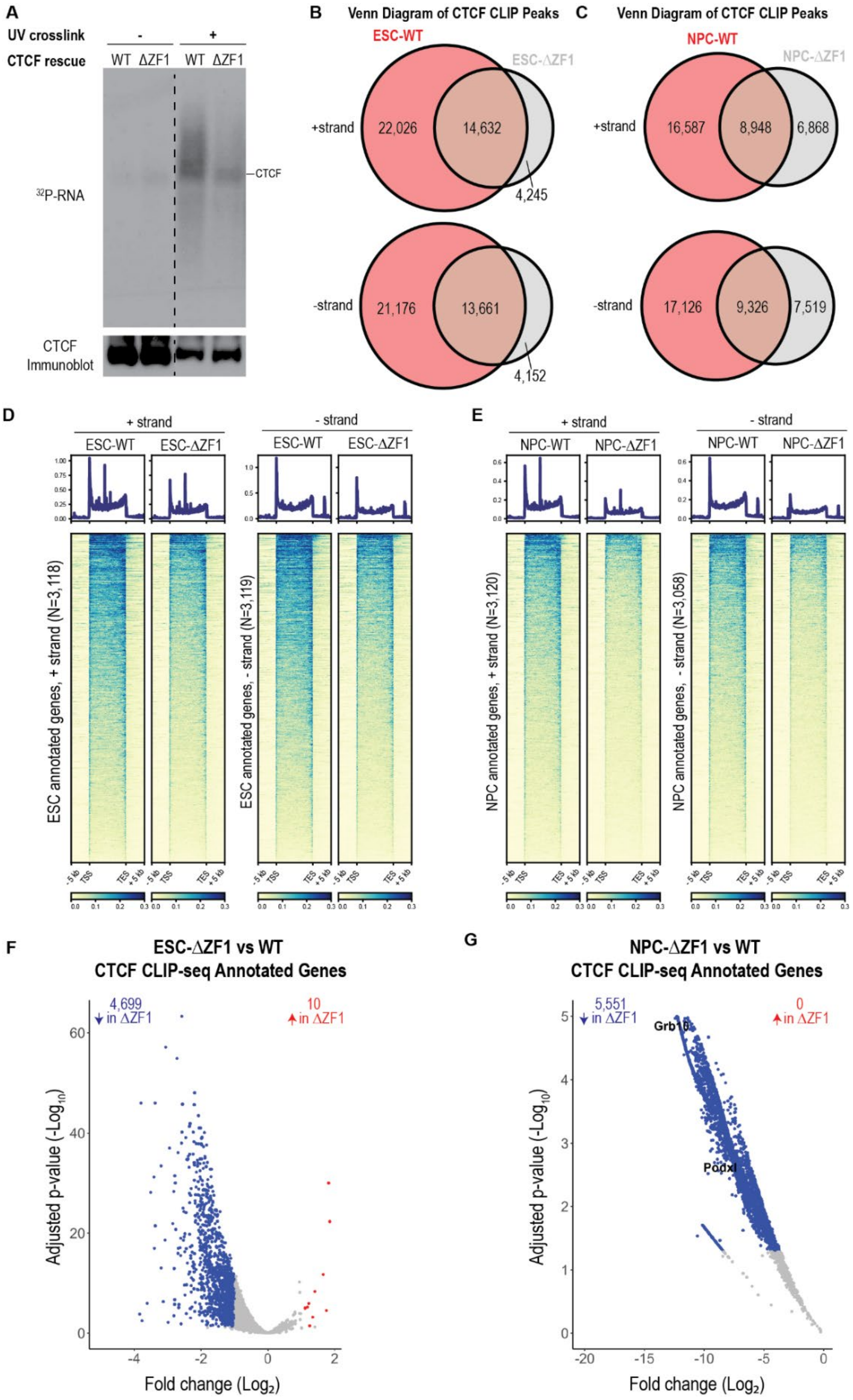

Fig. S9. CTCF-RNA interactions are decreased in ΔZF1. (**A**) UV crosslinking and radiolabeling of RNA co-immunoprecipitated with CTCF. Flag–Halo-tagged versions of CTCF, either WT or ΔZF1, were immunoprecipitated using anti-Flag beads under both non-crosslinked and UV-crosslinked conditions. RNA isolated from the immunoprecipitates was labeled with [γ-32P] ATP, and protein–RNA complexes were separated by gel

electrophoresis and transferred to a nitrocellulose membrane. The membrane was exposed to film to detect radiolabeled RNA. A CTCF immunoblot was performed on the same membrane. Dashed line indicates removal of unused lanes. (**B, C**) Reproducible CLIP peaks were called independently for WT and ΔZF1 (see Methods). Venn diagrams show overlap of CLIP peaks called in WT and ΔZF1 ESCs (B) and NPCs (C). (**D, E**) CLIP-seq signal (Flag-CTCF) was annotated to genes and plotted for WT versus ΔZF1 in ESCs (D) and NPCs (E). (**F, G**) Volcano plots of differential CTCF–RNA interactions comparing ΔZF1 to WT in ESCs (F) and NPCs (G), based on total CLIP-seq read counts from transcription start site (TSS) to transcription end site (TES). Each dot represents a gene. Read counts were normalized to background (non-crosslinked controls) and differential analysis was performed using DESeq2 (adjusted p-value cut-off ≤ 0.05, $\log_2$ fold-change cut-off ≥ 1 or ≤ -1; N=2-3 biological replicates).

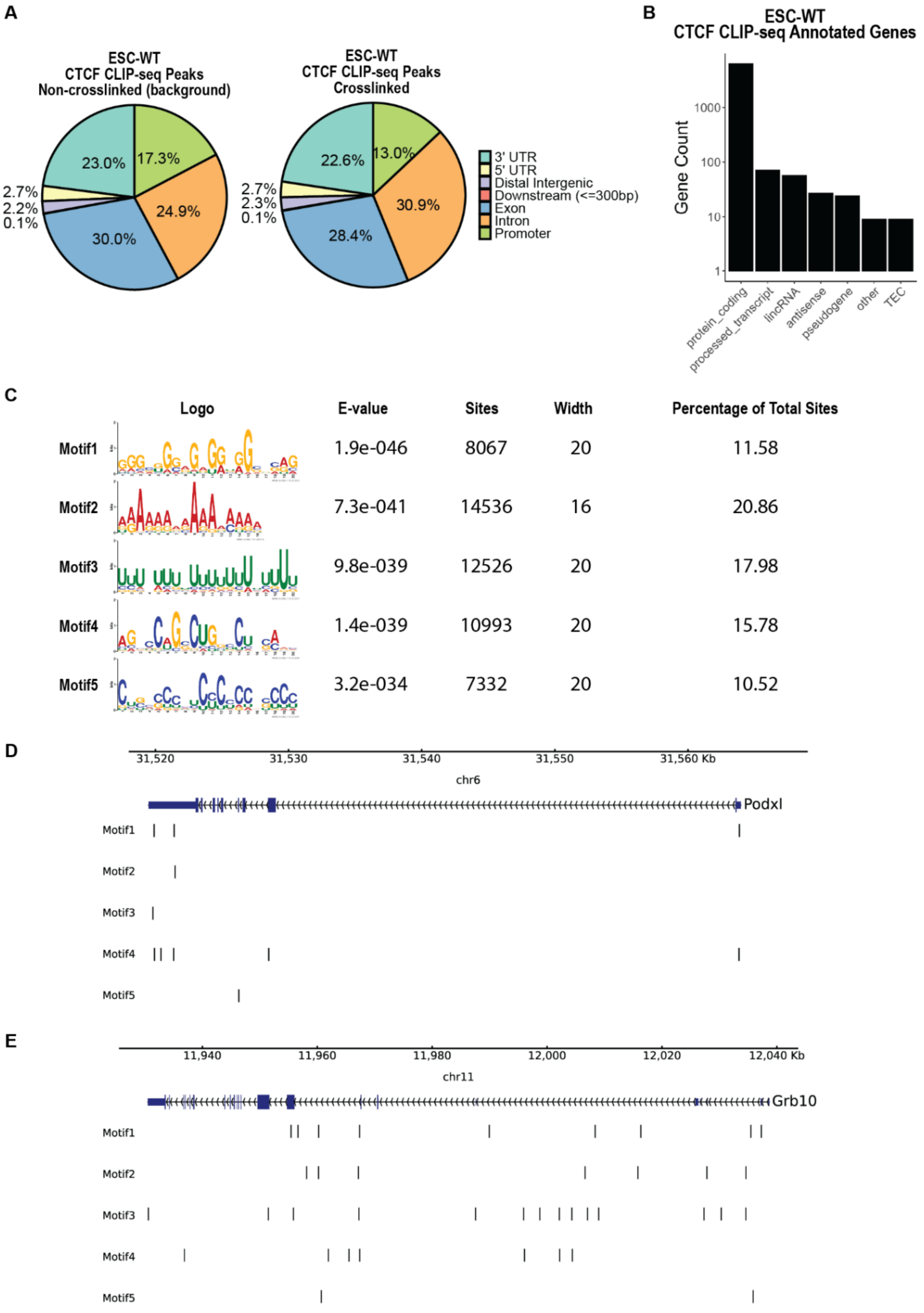

Fig. S10. Gene annotation and characteristics of CTCF CLIP-seq peaks. (**A**) CLIP peaks were identified in ESC-WT under both non-crosslinking (background) and UV-crosslinking conditions. Genomic annotations of the peaks were performed using ChIPseeker, and pie charts depict the distribution of peaks across genomic features (*e.g.*, exons,

introns, UTRs). (**B**) ESC-WT CLIP peaks were annotated to genes and categorized by gene type. Bar chart displays the number of annotated genes within each gene category. (**C**) *De novo* RNA motif discovery was performed on ESC-WT CLIP peaks using MEME Suite, with RNA-seq reads serving as the background. The analysis identified the top five enriched motifs. (**D**, **E**) The five motifs identified in (C) are mapped onto the *Podxl* (D) and *Grb10* (E) transcripts, showing their positions within each gene.

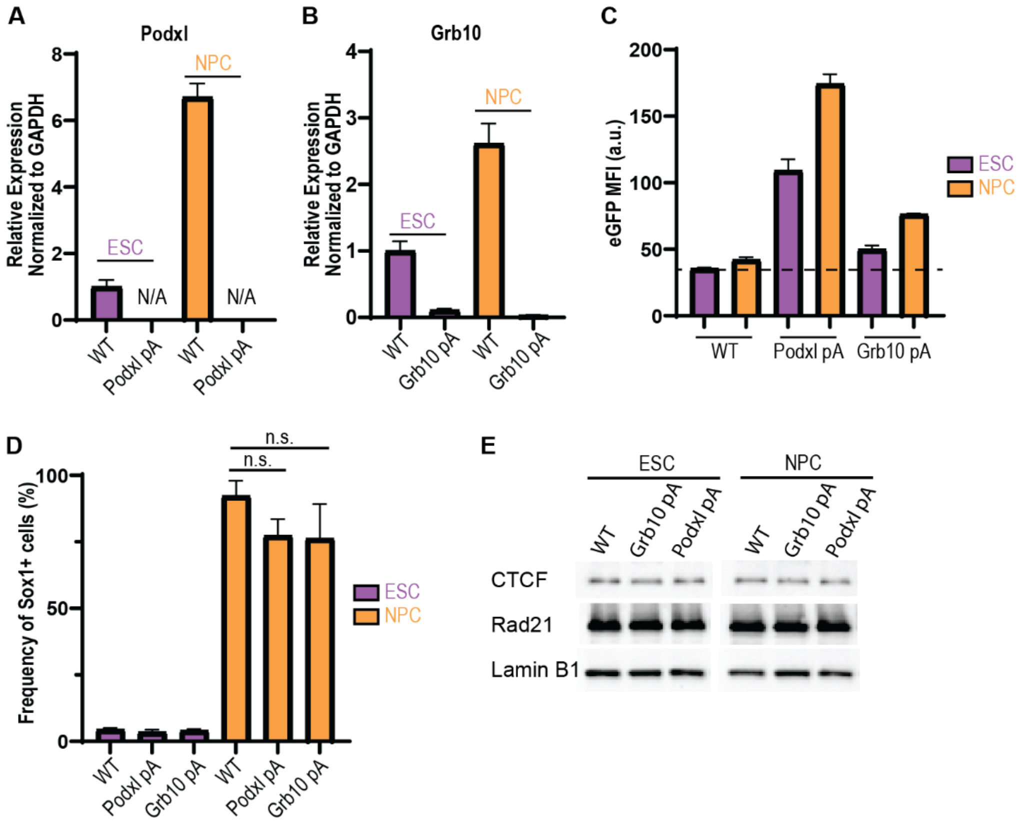

Fig. S11. Functional evaluation of Podxl-T2A-eGFP-SV40pA and Grb10-T2A-eGFP-SV40pA RNA-truncation mutants. (**A**, **B**) WT and (A) Podxl pA and (B) Grb10 pA cells were maintained as ESCs or differentiated into NPCs. RT-qPCR was done on (A) *Podxl* gene and (B) *Grb10* gene downstream of the pA insertion and normalized with GAPDH. Data are represented as mean ± SEM, N=3 biological replicates, N/A: Cq values > 40. (**C**) eGFP fluorescence of the Podxl pA and Grb10 pA mutants were analyzed by flow cytometry. Bar plots show mean fluorescence intensity (MFI, arbitrary units) of eGFP in the parental WT, Podxl pA, and Grb10 pA clones. Data are represented as mean ± SEM, N=3 biological replicates. Dashed line is the baseline fluorescence for eGFP-negative WT cells. (**D**) NPC differentiation was assessed in the pA mutants in comparison to the parental WT cells. Cells were fixed and immunostained with anti-Sox1 antibody. The percentages of Sox1+ cells were then analyzed by flow cytometry. Data are represented as mean ± SEM, p-values were determined using Dunnett's multiple comparison test, n.s.=not significant, N=3 biological replicates. (**E**) Western blot of CTCF and Rad21 in WT, Grb10 pA, and Podxl pA cells.

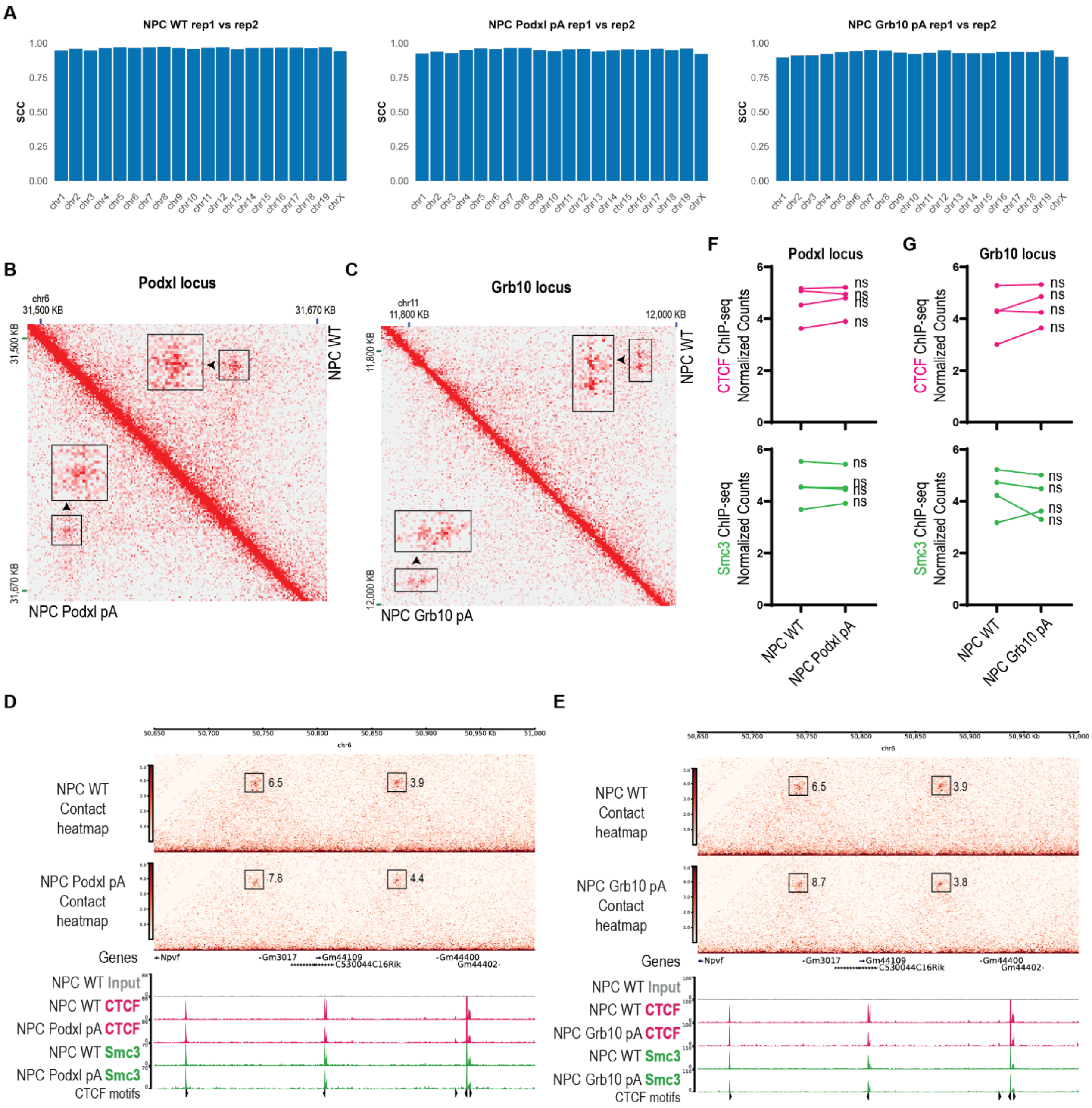

Fig. S12. Characterization of Podxl pA and Grb10 pA RNA-truncation mutants. (**A**) HicRep analysis showing stratum-adjusted correlation coefficients (SCC) between replicates of NPC WT and RNA-truncation mutants. Replicates of the same condition (WT, *Podxl* pA, or *Grb10* pA) exhibit high reproducibility, with SCC > 0.9. (**B**, **C**) Micro-C heatmaps were generated using VC_SQRT (Square root of Vanilla Coverage) normalization implemented in Juicer. Heatmaps compare WT to the Podxl pA mutant at the *Podxl* locus (B) and to the Grb10 pA mutant at the *Grb10* locus (C). Boxes indicate loop anchors and are enlarged for clarity. Data shown as merged biological replicates (N=2) (**D**, **E**) Micro-C contact heatmaps (top) and ChIP-seq tracks for CTCF and Smc3 (bottom) at a control locus distant from *Podxl* and *Grb10*. NPC WT is compared to either the Podxl pA mutant (D) or the Grb10 pA mutant (E). Boxes on the heatmaps denote loop anchors. Numbers next to each box indicate the fold enrichment of pixel intensity at the corresponding anchor relative to the local background, calculated using Cooltools. CTCF motif positions and orientations are annotated. Data are shown as merged biological replicates (Micro-C N=2; CTCF ChIP-seq N=2, Smc3 ChIP-seq N=2). (**F**, **G**) Dot plots showing DiffBind DESeq2-normalized ChIP-seq counts for CTCF and Smc3 at peaks overlapping the *Podxl* (F) and *Grb10* (G) loop anchors. Each dot represents a peak, with the counts representing the mean of 2 biological replicates. Lines connect the same peak between WT and RNA-truncation mutants. Statistical significance calculated using DiffBind. ns = not significant (FDR ≥ 0.05).

Table S1. ChIP-MS analysis of ESC-ZF1 mutant vs ESC-WT, related to **Fig. S7A**.
(Separate Excel file)

Table S2. ChIP-MS analysis of ESC-ZF10 mutant vs ESC-WT, related to **Fig. S7B**.
(Separate Excel file)

Table S3. RNA-seq analysis of ESC-ZF1 mutant vs ESC-WT, related to **Fig. S8A**.
(Separate Excel file)

Table S4. RNA-seq analysis of NPC-ZF1 mutant vs NPC-WT, related to **Fig. 4A**.
(Separate Excel file)

Table S5. Panther GO Term Enrichment of dysregulated genes in NPC-ZF1 mutant, related to **Fig. 4B**.
(Separate Excel file)

Table S6. Public datasets used in this study, related to **Fig. 4G**, **4H**, **S3J**, **S8F**.

**Cell line**	**Assay type**	**Target**	**Database**	**Experiment ID**
Mus musculus strain 129/Ola ES-E14	ChIP-seq	H3K27ac	ENCODE	ENCSR000CGQ
Mus musculus strain C57/BL-6 ES-derived neural progenitor cells	ChIP-seq	H3K27ac	NCBI	GSM2535239
Mus musculus strain C57/BL-6 ES-derived neural progenitor cells	ChIP-seq	H3K27ac	NCBI	GSM2535240
Mus musculus strain 129/Ola ES-E14	ATAC-seq	N/A	NCBI	GSE175902
Mus musculus strain 129/Ola ES-E14	ChIP-seq	H3K4me3	ENCODE	ENCSR000CGO
Mus musculus strain 129/Ola ES-E14	ChIP-seq	H3K27me3	ENCODE	ENCSR059MBO
Mus musculus strain 129/Ola ES-E14	ChIP-seq	H3K9me3	ENCODE	ENCSR000ADM

Table S7. CLIP-seq annotated genes in NPCs vs ESCs, related to **Fig. 6A**.
(Separate Excel file)

Table S8. Micro-C read depth, related to **Fig, 2**, **7**, **S4**, **S5**, **S6**, and **Methods**.
(Separate Excel file)

Table S9. Oligonucleotides used in this study, related to **Fig. 1A**, **S1B**, **S2A**, **S11A**, **S11B**, and **Methods**.
(Separate Excel file)

Table S10. Antibodies used in this study, related to **Fig. 1B**, **1C**, **3**, **S1C**, **S6**, **S7**, **S9A**, **S11D**, **S11E**, and **Methods**.

**Primary antibodies**	**Company**	**Catalogue #**	**Application**	**Dilutions**
Rabbit Anti-CTCF	Abcam	ab70303	ChIP, WB	ChIP: 2 ug/300 ug chromatin, WB: 1:5,000
Mouse Anti-Flag	Sigma	F1804	WB	1:500
Rabbit Anti-Lamin B1	Abcam	ab16048	WB	1:20,000
Goat Anti-Sox1	R&D Systems	AF3369	Flow cytometry	1:200 from 500 ug/mL reconstituted stock
Rabbit Anti-Rad21	Abcam	ab217678	ChIP, WB	ChIP: 4 ug/300 ug chromatin, WB: 1:5,000
Rabbit Anti-Smc3	Abcam	ab9263	ChIP	2 ug/300 ug chromatin
Mouse Anti-Oct3/4	Santa Cruz	sc-5279	WB	1:10,000
**Secondary antibodies**	**Company**	**Catalogue #**	**Application**	**Dilutions**
Donkey Anti-Goat IgG (H+L) Highly Cross-Adsorbed Secondary Antibody, Alexa Fluor™ Plus 647	Invitrogen	A32849	Flow cytometry	1:1,000
Goat Anti-Rabbit IgG (H+L)-HRP Conjugate	Bio-Rad	1706515	WB	1:10,000
Goat Anti-Mouse IgG (H+L)-HRP Conjugate	Bio-Rad	1706516	WB	1:5,000
